# State Minimum Wage and Mental Health Among Children and Adolescents

**DOI:** 10.1001/jamanetworkopen.2024.40810

**Published:** 2024-10-23

**Authors:** Nolan M. Kavanagh, Margaret McConnell, Natalie Slopen

**Affiliations:** 1 Interfaculty Initiative in Health Policy, Harvard University, Cambridge, Massachusetts; 2 Perelman School of Medicine, University of Pennsylvania, Philadelphia; 3 Harvard T.H. Chan School of Public Health, Boston, Massachusetts; 4 Center on the Developing Child, Harvard University, Cambridge, Massachusetts

## Abstract

**Importance:**

Mental health disorders are increasing for children and adolescents in the US, with those in poverty having especially high rates. More evidence is needed about the association between economic policies, such as the minimum wage, and children’s mental health.

**Objective:**

To test the association between minimum wages and the mental health of children and adolescents.

**Design, Setting, and Participants:**

This repeated cross-sectional, population-based US study used data from 2001 to 2022. Participants included state-representative, stratified random samples of children from the National Survey of Children’s Health and adolescents from the Youth Risk Behavior Surveillance System. Data analysis was performed from January 2023 to August 2024.

**Exposure:**

State minimum wage policies.

**Main Outcomes and Measures:**

A total of 15 outcomes reported by caregivers or adolescents from survey data were evaluated: depression, anxiety, attention deficit disorder and/or attention-deficit/hyperactivity disorder, behavior disorders, mood symptoms, gastrointestinal symptoms, considered and attempted suicide, any unmet health care and mental health care, alcohol and marijuana use, physical fights, school absenteeism, and paid employment. The associations between minimum wage policies and mental health were tested using 2-way fixed-effects models, lifetime minimum wage models, and difference-in-differences models with event studies, all with state, year, and birth cohort fixed effects. Additional controls included individual-level demographics and state-level health insurance and welfare policies.

**Results:**

Analyses included 239 534 children (aged 3-17 years; 117 111 girls [48.9%]) from the 2016 to 2022 National Survey of Children’s Health, and 1 453 043 adolescents (aged 12-18 years; 711 380 girls [49.0%]) from the 2001 to 2021 Youth Risk Behavior Surveillance System. Across diverse modeling approaches, increases in minimum wages were not associated with clear, consistent improvements in mental health. For example, in 2-way fixed-effects models, a $1 increase in the minimum wage was not associated with changes in rates of depression (0.06 percentage points [pp]; 95% CI, –0.11 to 0.23 pp; *P* = .48), unmet mental health care (0.13 pp; 95% CI, –0.04 to 0.30 pp; *P* = .14), or attempted suicide (–0.17 pp; 95% CI, –0.47 to 0.13 pp; *P* = .26). There also were no clear improvements when stratifying by household income, parental education, race and ethnicity, nativity, or urbanicity.

**Conclusions and Relevance:**

In this repeated cross-sectional study, changes in minimum wage policies over the past 2 decades were not accompanied by clear improvements in children’s and adolescents’ mental health. More evidence is needed on policy approaches to improve the mental well-being of children, particularly those from economically disadvantaged families.

## Key Points

**Question:**

What was the association between state minimum wage policies and the mental health of children and adolescents in the US over the past 2 decades?

**Findings:**

In this repeated cross-sectional study using 2 surveillance surveys of nearly 1.7 million children and adolescents from 2001 to 2022, there was no clear, compelling evidence that minimum wage increases were associated with improvements in 15 mental health outcomes, including diagnoses, symptoms, suicidality, substance use, health care access, school attendance, and work.

**Meaning:**

Recent increases in the minimum wage in the US have not been mirrored by improvements in children’s and adolescents’ mental health, suggesting that more evidence is needed on economic policies that can improve the mental health of children, especially those from economically disadvantaged families.

## Teaser

This cross-sectional study examines the association between minimum wages and the mental health of children and adolescents in the US.

## Introduction

Children and adolescents in the US are facing a mental health crisis.^1,2,3,4,5,6^ Mood and anxiety disorders are increasing, with 3% of children aged 3 to 17 years reporting depression and 9% reporting anxiety from 2016 to 2019.^7^ The COVID-19 pandemic has only accelerated the crisis.^8^ Poor mental health has pervasive impacts on a child’s quality of life and academic performance.^9^ Many of its consequences even last into adulthood, as adolescent mood disorders have been associated with lower long-run educational attainment, lower employment, and more chronic diseases.^10,11^

Poverty places an additional burden on families,^12^ with children in poverty having especially high rates of mental health disorders.^13^ Economic policies, such as increasing the minimum wage, have the potential to improve children’s mental health.^14,15^ Evidence suggests that increasing the minimum wage improves children’s physical health, including birth weights,^16^ infant mortality,^16,17^ school absenteeism,^18^ teen pregnancy rates,^19^ and indexes of overall health,^18^ especially for certain demographic groups.^20,21^ However, although the impact of minimum wages on adults’ mental health has been studied,^22,23,24,25,26,27^ limited work has examined the impact on that of children.^28,29,30^

Children’s mental health may be especially responsive to increasing minimum wages. A child’s emotional and behavioral problems tend to worsen with household economic stress,^13,31,32^ and increased wages might help alleviate it.^33^ For children with jobs, a change in the minimum wage might directly affect their mental health if it alters their employment status or earnings.^34^ For children in lower-income households more generally, higher earnings might allow caregivers to invest more time and resources into their children,^35,36,37^ identify and address mental health needs,^13,38^ or gain access to other health-promoting resources, such as better housing or schools.^39,40^

In this study, we used 2 national surveys that together included nearly 1.7 million children and adolescents in the US from 2001 to 2022 to test the association between minimum wage policies and children’s mental health. We applied several modeling approaches to 15 outcomes that collectively captured the clinical, behavioral, and social facets of mental well-being, including symptoms, diagnoses, substance use, health care access, school absenteeism, and work.

## Methods

### Study Populations

This cross-sectional study did not require institutional review board approval or informed consent because it used publicly available, deidentified data, in accordance with 45 CFR §46. We followed Strengthening the Reporting of Observational Studies in Epidemiology (STROBE) reporting guidelines for cross-sectional studies. We used 2 surveillance surveys of children’s health in the US: the 2016 to 2022 waves of the National Survey of Children’s Health (NSCH) and the 2001 to 2021 waves of the Youth Risk Behavior Surveillance System (YRBSS). Each survey captured a different period, target population, and outcomes of interest. The NSCH is a yearly, probability-based, national study of children’s physical and emotional well-being. It samples households known or projected to have a child according to US Census data; then, parents or guardians report on 1 of their children. Meanwhile, the YRBSS is a set of biennial, probability-based, state-level surveys of adolescents’ mental health and risk behaviors. As a school-based study, it samples classrooms in randomly selected high schools; then, adolescents directly respond to the surveys. State participation in the YRBBS is detailed in eAppendix 1 and eTable 1 in Supplement 1.

Consistent with surveillance studies that track mood disorders starting at age 3 years,^7^ we excluded all children younger than 3 years. We also excluded respondents without information for at least 1 outcome or their age (which was necessary to construct fixed effects). Respondents missing information for any given outcome were dropped pairwise from those analyses. All NSCH respondents had data on age and at least 1 outcome; depending on the outcome, pairwise missingness ranged from an unweighted 0.3% to 2.2%. Meanwhile, 0.5% of YRBSS respondents were excluded owing to missing age or all outcomes; pairwise missingness ranged from 2.5% to 24.3% by outcome, although most missingness was related to states that did not field all questions in all years. Complete numbers for each analysis are presented in the eFigures and eTables in Supplement 1. All analyses were weighted to represent all children or all students in grades 9 to 12 by state and year.

### Exposure and Outcome Measures

Our exposure was a state’s minimum wage in nominal US dollars. We used data from the US Department of Labor and took the higher of the state or federal minimum wage on January 1 of each year (eAppendix 2 and eFigure 1 in Supplement 1). As outcomes, we examined 15 dichotomous measures that collectively captured the clinical, behavioral, and social facets of children’s mental well-being. Some, including symptoms and coping mechanisms, should be especially sensitive to changes in financial stress.^13,31^ The exact wording and coding of all survey questions are provided in eAppendix 3 and eTable 2 in Supplement 1.

For the NSCH, all outcomes were reported by caregivers. We evaluated whether a child had any of 4 conditions identified by a health care practitioner and affecting the child at the time of the survey: (1) depression, (2) anxiety, (3) attention deficit disorder and/or attention-deficit/hyperactivity disorder (ADD/ADHD), and (4) behavior problems. All 4 have been linked to poverty and financial stress.^31,32,41,42^ We also examined the rates of children with moderate or severe symptoms of each condition (eAppendix 8 in Supplement 1). In addition, we evaluated whether a child (5) had chronic difficulty digesting food (eg, gastrointestinal problems, constipation, or diarrhea) in the past year, a common manifestation of anxiety in children^43,44,45^; (6) had not received necessary health care of any kind in the past year, as mental health disorders can have somatic symptoms^43,46^; (7) had not received necessary mental health services, specifically, in the past year; (8) had missed 7 or more days of school due to illness or injury in the past year (for ages 6-17 years), a potential consequence of debilitating mental illness^47,48,49^; or (9) had participated in any paid work in the past year (ages 6-17 years), as increased minimum wages might decrease children’s employment, thereby impacting their mental health directly or blunting the potential indirect health benefits of higher earnings for working families.^34,50^

For the YRBSS, all outcomes were reported by adolescents. We evaluated whether an adolescent had (1) felt sad or hopeless for 2 weeks or longer in the past year, a potential sign of depression; (2) considered or (3) attempted suicide in the past year; used (4) alcohol or (5) marijuana in the past month, which are potential coping mechanisms for financial stress^51^; or (6) been in a physical fight in the past year, a marker of behavior dysregulation associated with income.^52^

### Statistical Analysis

Data analysis was performed from January 2023 to August 2024. To test the association between state minimum wages and children’s mental health, we used 3 modeling approaches predominant in the minimum wage literature: (1) standard 2-way fixed-effects (TWFE) models,^17,20,21,25,27^ (2) human capital models,^16,18^ and (3) difference-in-differences and event study models.^23^ We describe the first in detail in the main text, with the remainder briefly reviewed here and fully described in eAppendixes 9, 10, and 11 in Supplement 1.

#### Approach Number 1

As our main analyses, we used ordinary least squares, TWFE models with a continuous measure of a state’s minimum wage in each year as the exposure. These models estimated the association between a $1 increase in the minimum wage and the percentage-point (pp) change in the prevalence of each outcome (eAppendix 6, eTables 4 and 5, and eFigures 2 and 3 in Supplement 1). They allowed us to use all states and years of data. We included state fixed effects to account for time-invariant social and policy characteristics of each state, year fixed effects to account for time-variant national economic trends, and birth year fixed effects to account for distinct cohort experiences over 2 decades of data.

On the respondent level, the NSCH models were adjusted for each child’s reported age, sex, race and ethnicity, family structure, highest education of any adult in the household, and nativity. The YRBSS models had fewer available controls and were adjusted for reported age, sex, race and ethnicity, and grade in high school. Regarding race and ethnicity, in the NSCH, caregivers reported the child's race and ethnicity; in the YRBSS, adolescents self-reported them. We included data on race and ethnicity here because families with racially minoritized identities may experience interpersonal and structural racism that can affect earning potential and mental health. All models were adjusted for other time-variant state policies that might affect low-income families, including each state’s Medicaid income eligibility limits for children aged 1 to 5 years and 6 to 18 years, whether the state had an earned income tax credit (EITC), the state’s EITC as a percentage of the federal EITC, whether the state’s EITC was refundable, and the state’s maximum Temporary Assistance for Needy Families benefits for a family of 3 (detailed in eAppendix 4 and eTable 3 in Supplement 1).^16,18^

As sensitivity analyses, we evaluated whether the standard TWFE results were similar using (1) real minimum wages (ie, adjusted for inflation); (2) wages lagged by 1 year^53^; (3) estimations using logistic regression^38^; (4) level-log models, which tested relative (rather than absolute) changes in wages; and (5) alternative SE structures. See details in eAppendix 8 in Supplement 1.

Notably, our main models included households of all incomes, a design analogous to intention to treat. Although workers earning near the minimum wage were most likely to see their take-home pay increase when policies changed, higher earners may have experienced spillover wage growth.^54^ Even so, we examined several subgroups that were more likely to benefit from increasing wages: in the NSCH, (1) households earning less than 200% of the Federal Poverty Level; (2) households whose adults had no more than a high school education; (3) Black and Hispanic or Latino children; (4) first-generation immigrants (ie, the child was born outside the US) or second-generation immigrants (ie, the child was born in the US but at least 1 parent was born outside the US); (5) adolescents (aged 13-17 years), many of whom work minimum wage jobs; and (6) children living in nonurban areas (ie, outside the principal cities of metropolitan statistical areas); and in the YRBSS, Black and Hispanic or Latino adolescents (eAppendix 7 in Supplement 1).

#### Approach Number 2

To help address the concern that standard TWFE models only capture contemporaneous changes in health, we implemented lifetime minimum wage models. These models took a human capital approach for long-term investments in health and used the average minimum wage to which a child was likely exposed over their entire life.^16,18^ They are detailed in eAppendix 9 in Supplement 1.

#### Approach Number 3

To help address the potential biases of TWFE models when policies are implemented at different times,^55,56,57^ we estimated difference-in-differences models with event studies. These analyses used the YRBSS from 2011 to 2021 to compare a subset of states that changed their policies at the same time against control states. They are detailed in eAppendixes 10 and 11 in Supplement 1.

Each approach has strengths that help overcome the weaknesses of others, with some taking advantage of all data and variation in minimum wages (ie, TWFE and lifetime wage models), some estimating longer-run associations (ie, lifetime wage and difference-in-differences models), and some eliminating the potential biases of staggered policy implementation (ie, difference-in-differences). Similarly, the data sources complement one another, with the NSCH allowing rich subgroup analyses and the YRBSS evaluating policy changes over 2 decades.

Given that we examined many outcomes, we implemented Bonferroni corrections for multiple hypothesis testing on all analyses in Supplement 1, as described in eAppendix 5. Statistical significance was set at 2-sided *P* < .05. All models included survey weights and clustered SEs by state. All analyses were performed in R statistical software version 4.3.1 (R Project for Statistical Computing).

## Results

### Approach Number 1: TWFE Models 

#### National Survey of Children’s Health

From 2016 to 2022, our analyses included 239 534 children in the NSCH (aged 3-17 years; 117 111 girls [48.9%]) (Table 1). In total, a weighted 9020 of 238 705 children (3.8%) reported having depression at the time of the survey, 20 430 of 238 501 children (8.6%) had anxiety, 21 766 of 237 222 children (9.2%) had ADD/ADHD, and 17 230 of 238 761 children (7.2%) had behavior problems. In the past year, 18 446 of 237 924 children (7.8%) reported chronic digestive issues, 8806 of 238 145 children (3.7%) had not received necessary medical care of any kind, 2861 of 238 145 children (1.2%) had not received necessary mental health care, 20 247 of 189 816 children (10.7%) had missed 7 or more days of school, and 39 536 of 187 581 children (21.1%) had some form of employment. During this period, minimum wages ranged from $7.25 to $16.10 across states and Washington, DC (eAppendix 2 in Supplement 1). Twenty-eight states plus Washington, DC, increased their wages at least once, with within-state variation ranging from $0.59 to $5.25, whereas 22 states never changed their policies.

**Table 1.** Demographic Characteristics of Children in the National Survey of Children’s Health From 2016 to 2022^a^

Characteristic	Participants, No. (%)	
	Unweighted (n = 239 534)	Weighted (n = 239 534)
Age, mean (SD) [range], y^b^	10.4 (4.5) [3-17]	10.1 (4.3) [3-17]
Sex		
Male	123 907 (51.7)	122 423 (51.1)
Female	115 627 (48.3)	117 111 (48.9)
Race and ethnicity		
American Indian or Alaska Native	1469 (0.6)	998 (0.4)
Asian, Native Hawaiian, or Pacific Islander	13 863 (5.8)	11 537 (4.8)
Black or African American	15 545 (6.5)	32 328 (13.5)
Hispanic or Latino	30 948 (12.9)	61 789 (25.8)
White	161 164 (67.3)	119 454 (49.9)
Other or ≥2 races^c^	16 545 (6.9)	13 428 (5.6)
Family structure		
Two parents, married	164 837 (68.8)	149 816 (62.5)
Two parents, not married	13 775 (5.8)	17 608 (7.4)
Single parent	45 988 (19.2)	52 041 (21.7)
Another family structure	9491 (4.0)	12 814 (5.3)
Not provided	5443 (2.3)	7255 (3.0)
Highest education of any adult in household		
Less than high school	6211 (2.6)	23 042 (9.6)
High school (including vocational or similar)	31 742 (13.3)	46 988 (19.6)
Some college or associate degree	54 228 (22.6)	50 976 (21.3)
College degree or higher	146 311 (61.1)	117 378 (49.0)
Not provided	1042 (0.4)	1151 (0.5)
Household nativity		
First generation (child was born outside the US)	4579 (1.9)	7818 (3.3)
Second generation (at least 1 parent was born outside the US)	37 701 (15.7)	54 622 (22.8)
Third generation or higher (both parents were born in the US)	180 632 (75.4)	155 525 (64.9)
Not provided	16 622 (6.9)	21 569 (9.0)

^a^ Estimates with and without survey weights are provided.

^b^ Age is presented as continuous but treated as categorical in all models.

^c^ Other refers to any other race or ethnicity not already specified.

**Figure 1.**
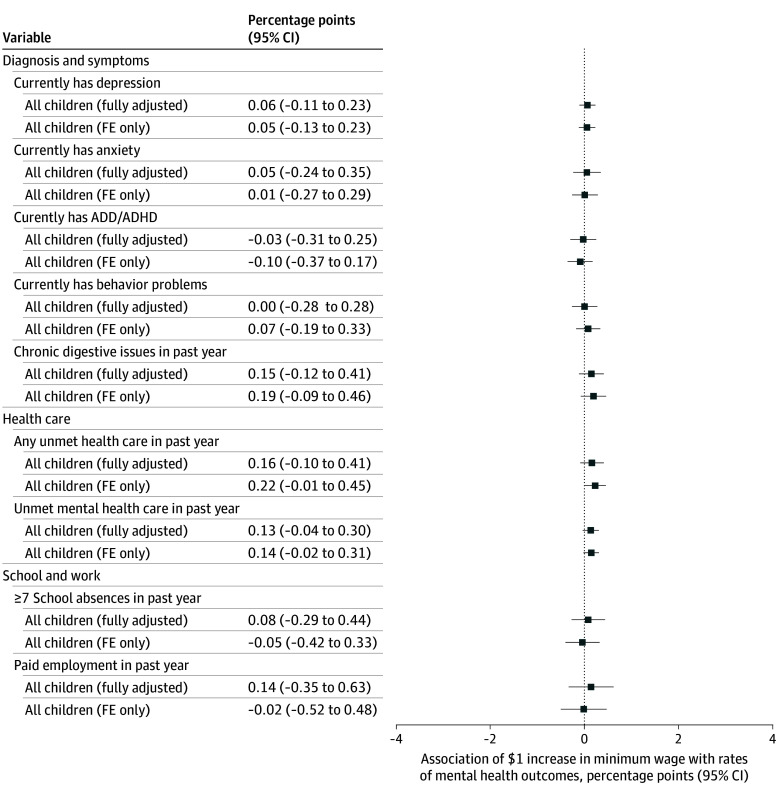
Associations Between State Minimum Wages and the Mental Health of Children, Aged 3 to 17 Years, 2016 to 2022 The coefficients provide the percentage-point association between a $1 increase in a state’s minimum wage and the prevalence of each outcome. Negative values represent improvements in the mental health of the population. Estimates are based on 2-way fixed-effects (FE) models with children aged 3 to 17 years in the National Survey of Children’s Health from 2016 to 2022 (except for absenteeism and employment, which were only asked of children aged 6-17 years). All models were adjusted for state, year, and birth cohort FE. Fully adjusted models also controlled for each child’s reported age, sex, race and ethnicity, family structure, parental education, and nativity, as well as state-level Medicaid income eligibility limits, Earned Income Tax Credit policies, and Temporary Assistance for Needy Families benefits for families of 3 (see methods for full details). SEs were clustered at the state level; 95% CIs are provided. Full regression results are provided in eTable 4 in Supplement 1. ADD/ADHD indicates attention deficit disorder and/or attention-deficit/hyperactivity disorder.

However, increased minimum wages from 2016 to 2022 were not associated with significant improvements in children’s mental health for any outcome in adjusted TWFE analyses, including depression (0.06 pp; 95% CI, –0.11 to 0.23 pp; *P* = .48), anxiety (0.05 pp; 95% CI, –0.24 to 0.35 pp; *P* = .72), ADD/ADHD (–0.03 pp; 95% CI, –0.31 to 0.25 pp; *P* = .84), behavior problems (0.00 pp; 95% CI, –0.28 to 0.28 pp; *P* = .99), digestive issues (0.15 pp; 95% CI, –0.12 to 0.41 pp; *P* = .28), any unmet health care (0.16 pp; 95% CI, –0.10 to 0.41 pp; *P* = .22), unmet mental health care (0.13 pp; 95% CI, –0.04 to 0.30 pp; *P* = .14), school absenteeism (0.08 pp; 95% CI, –0.29 to 0.44 pp; *P* = .67), and paid employment (0.14 pp; 95% CI, –0.35 to 0.63 pp; *P* = .57) (Figure 1). For all outcomes, the adjusted 95% CIs ruled out an improvement of 0.4 pp or greater per $1 increase in the minimum wage.

In subgroup analyses, there was little evidence of benefits for children living in households with incomes less than 200% of the Federal Poverty Level, households with no more than a high school education, Black and Hispanic or Latino children, first-generation or second-generation immigrants, adolescents, and children in nonurban areas (eAppendix 7, eTable 6, and eFigures 4 and 5 in Supplement 1). There was generally null, otherwise conflicting evidence in several sensitivity analyses, including inflation-adjusted wages, lagged wages, and symptom severity (eAppendix 8 and eFigures 6-14 in Supplement 1).

#### Youth Risk Behavior Surveillance System

From 2001 to 2021, our analyses included 1 453 043 adolescents in the YRBSS (aged 12-18 years; 711 380 girls [49.0%]) (Table 2). In the past year, a weighted 433 063 of 1 416 604 adolescents (30.6%) reported being sad or hopeless for 2 or more weeks, 237 429 of 1 418 760 (16.7%) considered suicide, 109 106 of 1 213 412 (9.0%) attempted suicide, and 349 295 of 1 345 461 (26.0%) had been in a physical fight. In the past month, 446 011 of 1 309 507 adolescents (34.1%) reported using alcohol and 270 708 of 1 401 100 (19.3%) used marijuana. During this period, minimum wages ranged from $5.15 to $13 across the 45 included states. Since the federal minimum wage increased from $5.15 to $7.25 between 2008 and 2010, all states experienced at least 1 increase in their minimum wages, with within-state variation ranging from $2.10 to $7.35 (eAppendix 2 in Supplement 1).

**Table 2.** Demographic Characteristics of Adolescents in the Youth Risk Behavior Surveillance System From 2001 to 2021^a^

Characteristic	Participants, No. (%)	
	Unweighted (n = 1 453 043)	Weighted (n = 1 453 043)
Age, y		
≤12	6072 (0.4)	4552 (0.3)
13	6158 (0.4)	4264 (0.3)
14	206 942 (14.2)	169 189 (11.6)
15	386 597 (26.6)	370 198 (25.5)
16	379 972 (26.2)	372 401 (25.6)
17	321 745 (22.1)	338 973 (23.3)
≥18	145 557 (10.0)	193 465 (13.3)
Sex		
Male	711 254 (48.9)	734 991 (50.6)
Female	732 927 (50.4)	711 380 (49.0)
Not provided	8862 (0.6)	6672 (0.5)
Race and ethnicity		
American Indian or Alaska Native	31 881 (2.2)	19 076 (1.3)
Asian, Native Hawaiian, or Pacific Islander	79 962 (5.5)	51 140 (3.5)
Black or African American	188 827 (13.0)	242 602 (16.7)
Hispanic or Latino	245 565 (16.9)	282 826 (19.5)
White	804 296 (55.4)	790 176 (54.4)
Multiple races, non-Hispanic	68 347 (4.7)	38 200 (2.6)
Not provided	34 165 (2.4)	29 022 (2.0)
School grade		
9th	412 224 (28.4)	403 189 (27.7)
10th	388 849 (26.8)	371 600 (25.6)
11th	348 665 (24.0)	341 545 (23.5)
12th	286 358 (19.7)	324 043 (22.3)
Not provided	16 947 (1.2)	12 666 (0.9)

^a^ Estimates with and without survey weights are provided.

**Figure 2.**
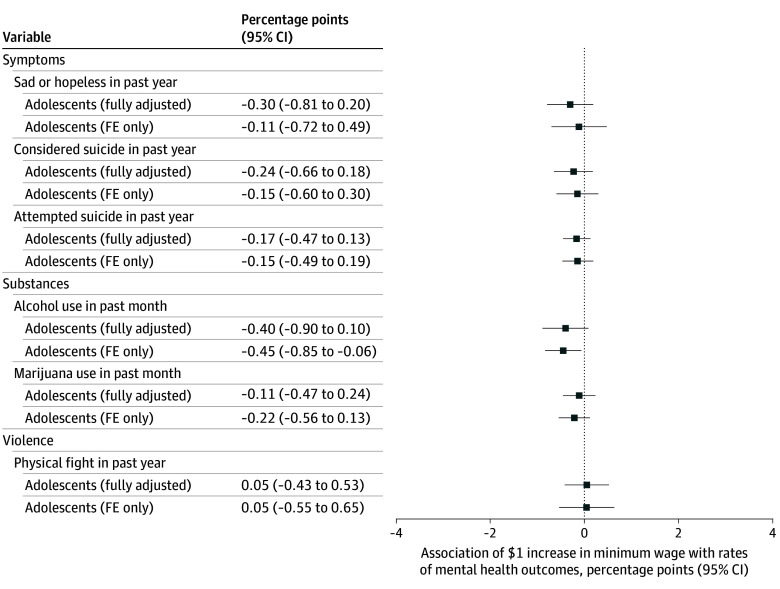
Associations Between State Minimum Wages and the Mental Health of Adolescents, Aged 12 to 18 Years, 2001 to 2021 The coefficients provide the percentage-point association between a $1 increase in a state’s minimum wage and the prevalence of each outcome. Negative values represent improvements in the mental health of the population. Estimates are based on 2-way fixed-effects (FE) models with adolescents aged 12 to 18 years in the Youth Risk Behavior Surveillance System from 2001 to 2021. All models were adjusted for state, age, and birth cohort FE. Fully adjusted models also controlled for each adolescent’s reported age, sex, race and ethnicity, and grade in high school, as well as state-level Medicaid income eligibility limits, Earned Income Tax Credit policies, and Temporary Assistance for Needy Families benefits for families of 3 (see methods for full details). SEs were clustered at the state level; 95% CIs are provided. Full regression results are provided in eTable 5 in Supplement 1.

Even so, increased minimum wages from 2001 to 2021 were not associated with significant improvements in adolescents’ mental health for any outcome in adjusted TWFE analyses, including being sad or hopeless (–0.30 pp; 95% CI, –0.81 to 0.20 pp; *P* = .23), considering suicide (–0.24 pp; 95% CI, –0.66 to 0.18 pp; *P* = .26), attempting suicide (–0.17 pp; 95% CI, –0.47 to 0.13 pp; *P* = .26), using alcohol (–0.40 pp; 95% CI, –0.90 to 0.10 pp; *P* = .11), using marijuana (–0.11 pp; 95% CI, –0.47 to 0.24 pp; *P* = .53), or fighting (0.05 pp; 95% CI, –0.43 to 0.53 pp; *P* = .84) (Figure 2). For all outcomes, the adjusted 95% CIs ruled out an improvement of 0.9 pp or greater per $1 increase in the minimum wage. Similarly, there was no evidence of benefits for Black and Hispanic or Latino adolescents (eAppendix 7 in Supplement 1), and generally null, otherwise conflicting evidence using inflation-adjusted wages, lagged wages, or several other sensitivity analyses (eAppendix 8 in Supplement 1).

### Approach Number 2: Lifetime Minimum Wage Models

The lifetime minimum wage models provided largely null, otherwise conflicting evidence for the association between long-term exposure to minimum wage policies and children’s mental health (eAppendix 9 and eFigures 15 and 16 in Supplement 1). Some estimates were statistically significant at the 5% level, but they pointed in conflicting directions and none remained significant after Bonferroni corrections.

### Approach Number 3: Difference-in-Differences Models

The difference-in-differences models provided little evidence for a longer-run association between increasing minimum wages and adolescents’ mental health (eAppendixes 10 and 11, eFigures 17-21, and eTables 7 and 8 in Supplement 1). Sporadic event study coefficients were statistically significant, but they were misaligned with policy changes and lacked the steadily growing associations expected of steadily increasing minimum wages in treated states.

## Discussion

This national cross-sectional study did not find clear, compelling evidence that minimum wage increases in the US over the past 2 decades were associated with improvements in children’s and adolescents’ mental health. Using 2 national surveys, 15 multifaceted outcomes, and several modeling approaches to carefully test the association, it retrieved generally null, otherwise conflicting associations. The study also did not find clear evidence of benefit for several disadvantaged subgroups, including lower-income, immigrant, and racially minoritized children.

Existing work on the minimum wage and mental health has largely focused on adults.^22^ In the US, increasing minimum wages have been associated with improved mental health for several populations,^25,26^ fewer stressful life events for pregnant persons,^27^ and decreased suicides.^53,58^ Mixed findings have been observed in Britain.^23,24^ Other economic policies, such as tax credits and cash transfers, have also improved the mental health of adults.^59,60,61^ Among children, recent work in the US has found evidence of beneficial associations between the minimum wage and limited mental health outcomes, including violent behaviors,^29^ youth homicide rates,^28^ and child maltreatment,^30^ often within specific subgroups. However, the current study did not find evidence of broader mental health benefits for US children.

Given the strong link between poverty and mental health,^13,31,32,41,42^ it is unclear why the current study did not find evidence of benefit. One concern might be offsetting forces. For example, increasing wages might reduce the true rate of depression while enabling families to seek medical care and get overdue diagnoses, thereby increasing the apparent rate. However, there was little evidence of improvement in any intermediate outcomes, including employment, symptoms, proxies for stress or coping, access to health care, and school attendance. Another concern might be that too few families earn near the minimum wage. However, in 2022, 52 million workers in the US earned less than $15 per hour, including 6 million teenagers and 11 million single parents.^62^

Instead, other structural barriers may have blunted the benefits of increasing wages. For example, many low-wage workers face benefits cliffs, or parallel reductions in public benefits that offset higher wages.^63^ In addition, increases in the minimum wage can (but not always) lead to reduced hours for low-wage workers.^63,64,65,66^ Although low-income children are less likely to work than other children, they tend to work more hours.^67^ This study did not find an association between minimum wages and children’s overall employment, but it did not observe whether children had their hours or benefits cut. Policymakers must take into account other economic and policy influences when increasing the minimum wage to ensure that working families see net benefits.

### Limitations

This study has several limitations. First, it only considered federal-level and state-level policies, not city-level or county-level policies. Several, mostly urban, localities have raised minimum wages above and beyond state law.^64,68,69^ It is possible that changes in local minimum wages during the past 2 decades produced meaningful improvements in children’s and adolescents’ mental health that this study did not capture. That said, when the economic effects of local minimum wages have been compared with those of state-level policies, the 2 estimates have tended to be similar.^70^ Moreover, this study did not find beneficial associations in subgroup analyses of children outside major US cities, who were unlikely to experience local minimum wage changes.

Second, this study was based on weighted survey data, which are vulnerable to sampling, response, and weighting biases. Even so, the results were similar using 2 national surveys with different sampling schemes and reports by both caregivers and adolescents, all of which help mitigate the risk of bias.^71^ Third, the study relied on several modeling approaches, each with its own weaknesses.^55,56,57^ Even so, none showed clear evidence of benefit. In addition, although many of the nulls were precisely estimated, they cannot exclude the possibility of more modest associations, nor can they speak to the potential consequences of more ambitious increases in the minimum wage.

## Conclusions

Taken together, these findings suggest that increases in the minimum wage over the past 2 decades in the US were not associated with meaningful improvements in children’s and adolescents’ mental health. Although there are many social, economic, and political reasons to increase the minimum wage,^22,72,73^ more evidence is needed on other policies to improve the mental health of children, especially in disadvantaged families.

## Notes

Supplement 1. **eAppendix 1.** State Participation in the YRBSS **eAppendix 2.** Coding of Exposure **eAppendix 3.** Coding of Outcomes **eAppendix 4.** Individual-Level and State Policy Controls **eAppendix 5.** Corrections for Multiple Comparisons **eAppendix 6.** Approach #1: Two-Way Fixed Effects (TWFE) Models **eAppendix 7.** Approach #1: TWFE Subgroup Analyses **eAppendix 8.** Approach #1: TWFE Sensitivity Analyses **eAppendix 9.** Approach #2: Lifetime Minimum Wage Models **eAppendix 10.** Approach #3: Difference-in-Differences and Event Study Models **eAppendix 11.** Approach #3: Event Study Sensitivity Analyses **eTable 1.** Unweighted Number of Respondents to the YRBSS by state and year **eTable 2.** Question Wording and Coding of All Mental Health Outcomes **eTable 3.** Control Variables Included in Minimum Wage Models **eTable 4.** Full Regression Results for the Standard TWFE Models in the NSCH **eTable 5.** Full Regression Results for the Standard TWFE Models in the YRBSS **eTable 6.** List of Subgroup Analyses for Standard TWFE Models **eTable 7.** Difference-in-Difference Models in the YRBSS From 2011–2021 **eTable 8.** States in Balanced Panels for Event Studies by Outcome **eFigure 1.** Effective Minimum Wages for Each State From 2001 to 2022 **eFigure 2.** Standard TWFE Models With Bonferroni Corrections in the NSCH **eFigure 3.** Standard TWFE Models With Bonferroni Corrections in the YRBSS **eFigure 4.** Subgroup TWFE Models in the NSCH **eFigure 5.** Subgroup TWFE Models in the YRBSS **eFigure 6.** TWFE Models With Lagged and Inflation-Adjusted Wages in the NSCH **eFigure 7.** TWFE Models With Lagged and Inflation-Adjusted Wages in the YRBSS **eFigure 8.** TWFE Models Using Logistic Regression in the NSCH **eFigure 9.** TWFE Models Using Logistic Regression in the YRBSS **eFigure 10.** Level-Log TWFE Models in the NSCH **eFigure 11.** Level-Log TWFE Models in the YRBSS **eFigure 12.** TWFE models using nested clusters in the NSCH **eFigure 13.** TWFE models using nested clusters in the YRBSS **eFigure 14.** TWFE models for symptom severity in the NSCH **eFigure 15.** Lifetime Minimum Wage Models in the NSCH **eFigure 16.** Lifetime Minimum Wage Models in the YRBSS **eFigure 17.** Treatment and Control States for the Difference-In-Differences Models **eFigure 18.** Weighted Mean Minimum Wage in Treatment States by Year **eFigure 19.** Main Event Studies in the YRBSS From 2011–2021 **eFigure 20.** Event Studies Using Nested Clusters in the YRBSS **eFigure 21.** Event Studies Using a Strictly Balanced Panel in the YRBSS
eReferences

Supplement 2.
Data Sharing Statement
