# Supplementary material for: State Minimum Wage and Mental Health Among Children and Adolescents
Source: JAMA Netw Open. 2024 Oct 23;7(10):e2440810. doi: 10.1001/jamanetworkopen.2024.40810 (PMC11581516; doi:10.1001/jamanetworkopen.2024.40810)
Supplement: Supplement 1. — eAppendix 1. State Participation in the YRBSS eAppendix 2. Coding of Exposure eAppendix 3. Coding of Outcomes eAppendix 4. Individual-Level and State Policy Controls eAppendix 5. Corrections for Multiple Comparisons eAppendix 6. Approach #1: Two-Way Fixed Effects (TWFE) Models eAppendix 7. Approach #1: TWFE Subgroup Analyses eAppendix 8. Approach #1: TWFE Sensitivity Analyses eAppendix 9. Approach #2: Lifetime Minimum Wage Models eAppendix 10. Approach #3: Difference-in-Differences and Event Study Models eAppendix 11. Approach #3: Event Study Sensitivity Analyses eTable 1. Unweighted Number of Respondents to the YRBSS by state and year eTable 2. Question Wording and Coding of All Mental Health Outcomes eTable 3. Control Variables Included in Minimum Wage Models eTable 4. Full Regression Results for the Standard TWFE Models in the NSCH eTable 5. Full Regression Results for the Standard TWFE Models in the YRBSS eTable 6. List of Subgroup Analyses for Standard TWFE Models eTable 7. Difference-in-Difference Models in the YRBSS From 2011–2021 eTable 8. States in Balanced Panels for Event Studies by Outcome eFigure 1. Effective Minimum Wages for Each State From 2001 to 2022 eFigure 2. Standard TWFE Models With Bonferroni Corrections in the NSCH eFigure 3. Standard TWFE Models With Bonferroni Corrections in the YRBSS eFigure 4. Subgroup TWFE Models in the NSCH eFigure 5. Subgroup TWFE Models in the YRBSS eFigure 6. TWFE Models With Lagged and Inflation-Adjusted Wages in the NSCH eFigure 7. TWFE Models With Lagged and Inflation-Adjusted Wages in the YRBSS eFigure 8. TWFE Models Using Logistic Regression in the NSCH eFigure 9. TWFE Models Using Logistic Regression in the YRBSS eFigure 10. Level-Log TWFE Models in the NSCH eFigure 11. Level-Log TWFE Models in the YRBSS eFigure 12. TWFE models using nested clusters in the NSCH eFigure 13. TWFE models using nested clusters in the YRBSS eFigure 14. TWFE models for symptom severity in the NSCH eFigure 15. Lifetime Minimum Wage Mo [file jamanetwopen-e2440810-s001.pdf]

Supplemental Online Content

Kavanagh NM, McConnell M, Slopen N. State minimum wage and mental health among children and adolescents. *JAMA Netw Open*. 2024;7(10):e2440810.
doi:10.1001/jamanetworkopen.2024.40810

eAppendix 1. State Participation in the YRBSS
eAppendix 2. Coding of Exposure
eAppendix 3. Coding of Outcomes
eAppendix 4. Individual-Level and State Policy Controls
eAppendix 5. Corrections for Multiple Comparisons
eAppendix 6. Approach #1: Two-Way Fixed Effects (TWFE) Models
eAppendix 7. Approach #1: TWFE Subgroup Analyses
eAppendix 8. Approach #1: TWFE Sensitivity Analyses
eAppendix 9. Approach #2: Lifetime Minimum Wage Models
eAppendix 10. Approach #3: Difference-in-Differences and Event Study Models
eAppendix 11. Approach #3: Event Study Sensitivity Analyses
eTable 1. Unweighted Number of Respondents to the YRBSS by State and Year
eTable 2. Question Wording and Coding of All Mental Health Outcomes
eTable 3. Control Variables Included in Minimum Wage Models
eTable 4. Full Regression Results for the Standard TWFE Models in the NSCH
eTable 5. Full Regression Results for the Standard TWFE Models in the YRBSS
eTable 6. List of Subgroup Analyses for Standard TWFE Models
eTable 7. Difference-in-Difference Models in the YRBSS From 2011–2021
eTable 8. States in Balanced Panels for Event Studies by Outcome
eFigure 1. Effective Minimum Wages for Each State From 2001 to 2022
eFigure 2. Standard TWFE Models With Bonferroni Corrections in the NSCH
eFigure 3. Standard TWFE Models With Bonferroni Corrections in the YRBSS
eFigure 4. Subgroup TWFE Models in the NSCH
eFigure 5. Subgroup TWFE Models in the YRBSS
eFigure 6. TWFE Models With Lagged and Inflation-Adjusted Wages in the NSCH
eFigure 7. TWFE Models With Lagged and Inflation-Adjusted Wages in the YRBSS
eFigure 8. TWFE Models Using Logistic Regression in the NSCH
eFigure 9. TWFE Models Using Logistic Regression in the YRBSS
eFigure 10. Level-Log TWFE Models in the NSCH
eFigure 11. Level-Log TWFE Models in the YRBSS
eFigure 12. TWFE Models Using Nested Clusters in the NSCH
eFigure 13. TWFE Models Using Nested Clusters in the YRBSS
eFigure 14. TWFE Models for Symptom Severity in the NSCH
eFigure 15. Lifetime Minimum Wage Models in the NSCH
eFigure 16. Lifetime Minimum Wage Models in the YRBSS
eFigure 17. Treatment and Control States for the Difference-in-Differences Models
eFigure 18. Weighted Mean Minimum Wage in Treatment States by Year
eFigure 19. Main Event Studies in the YRBSS from 2011–2021
eFigure 20. Event Studies Using Nested Clusters in the YRBSS
eFigure 21. Event Studies Using a Strictly Balanced Panel in the YRBSS
eReferences

This supplemental material has been provided by the authors to give readers additional information about their work.

© 2024 Kavanagh NM et al. *JAMA Network Open.*

eAppendix 1. State Participation in the YRBSS

Not all states participated in the YRBSS in all years. The following table presents the unweighted number of adolescents included in our analyses for each state-year. Note that some states did not ask all questions in all years and have lower Ns for those analyses.

eTable 1. Unweighted number of respondents to the YRBSS by state and year.

	2001	2003	2005	2007	2009	2011	2013	2015	2017	2019	2021
**AK**	0	1477	0	1314	1347	1327	1234	1412	1327	1867	0
**AL**	1576	1085	1130	0	1515	1357	1573	1565	0	2032	418
**AR**	1690	0	1588	1606	1685	1370	1540	2873	1675	2019	1547
**AZ**	0	3421	3298	3084	2589	2884	1621	2572	2136	1921	1180
**CA**	0	0	0	0	0	0	0	1936	1769	1332	0
**CO**	0	0	1492	0	1508	1483	0	0	1485	1344	1031
**CT**	0	0	2247	2066	2390	2053	2403	2397	2419	2004	1759
**DE**	2904	3028	2687	2595	2390	2279	2714	2742	2879	0	1566
**FL**	4222	4068	4545	4503	5598	6186	6061	6329	6141	5673	4665
**GA**	0	2062	1752	2460	1878	1967	1984	0	0	4541	604
**HI**	0	0	1660	1191	1508	4322	4622	6069	6015	5835	5630
**IA**	0	0	1359	1437	0	1530	0	0	1691	1587	1385
**ID**	1712	1730	1456	1439	2162	1698	1884	1756	1815	1208	989
**IL**	0	0	0	2434	3042	3605	3268	3274	4993	3118	2970
**IN**	0	1668	1528	2315	1514	2853	0	1909	0	0	1026
**KS**	0	0	1652	1724	2023	1873	1938	0	2411	1415	1512
**KY**	0	1607	3273	3590	1776	1736	1624	2569	1996	1992	2172
**LA**	0	0	0	1345	1031	1159	1103	0	1266	1301	634
**MD**	0	0	1410	1526	1643	2911	53498	55268	50770	40867	35450
**ME**	1348	1677	1370	1323	9178	9805	8960	9579	9477	8358	6500
**MI**	3624	3447	3248	3523	3406	4191	4264	4809	1622	4559	3741
**MO**	1649	1549	1877	1553	1622	0	1607	1493	1863	1216	892
**MS**	1801	1481	0	1606	1793	1827	1583	2146	0	1763	1741
**MT**	2733	2774	3068	4010	1849	4141	4882	4478	4733	3808	4463
**NC**	2545	2551	3870	3503	5698	2274	1845	6161	3145	3043	1718
**ND**	1599	1664	1724	1766	1834	1910	1978	2121	2140	2040	2007
**NE**	0	2932	3754	0	0	2725	1806	1682	1425	1324	676
**NH**	0	1325	1273	1637	1491	1404	1624	14656	11919	13607	13718
**NJ**	2139	0	1492	0	1756	1654	1700	0	0	1389	695
**NM**	0	0	5607	2629	5027	5869	5447	8297	5772	7593	4552

	2001	2003	2005	2007	2009	2011	2013	2015	2017	2019	2021
**NV**	1462	1982	1555	1783	2082	0	2128	1447	1666	1405	1708
**NY**	0	9301	9689	13406	14843	13172	10612	10792	11348	10808	4617
**OK**	0	1381	1712	2606	1413	1143	1474	1609	1642	2003	1684
**PA**	0	0	0	0	2077	0	0	2897	3751	2334	1635
**RI**	1390	1808	2349	2206	3209	3951	2453	3451	2214	1610	2148
**SC**	0	0	1307	1238	1106	1488	1605	1356	1498	1216	1601
**SD**	1613	1824	1586	1609	2167	1542	1316	1312	0	1453	944
**TN**	0	1937	1540	2062	2213	2631	1899	4121	2041	2222	1966
**TX**	7055	0	4125	3164	3498	4196	3174	0	2108	2031	1680
**UT**	1067	1447	1549	1974	1595	1707	2194	0	1845	1537	1499
**VA**	0	0	0	0	0	1438	6927	4413	3687	4602	3080
**VT**	7144	6009	7165	6680	9398	8579	0	20837	20475	18588	17373
**WI**	2117	2121	2385	2094	2431	3042	2839	0	2064	1826	1834
**WV**	0	1748	1367	1392	1665	2169	1789	1621	1560	1402	482
**WY**	2757	1549	2494	2237	2893	2516	3010	2420	0	0	0

eAppendix 2. Coding of Exposure

As our main exposure, we used a state's effective minimum wage, i.e. the higher of the state or federal minimum wage, not adjusted for inflation, on January 1 each year, based on data compiled by the U.S. Department of Labor (https://www.dol.gov/agencies/whd/state/minimum-wage/history) (**eFigure 1**). For states with several minimum wages (e.g. by industry or occupation), we used the lowest one. The range was $5.15 to $16.10. The federal minimum wage increased from $5.15 to $7.25 from 2008–2010. For any analyses with modifications to this measure, e.g. inflation adjustment, we describe those changes in the relevant sections below.

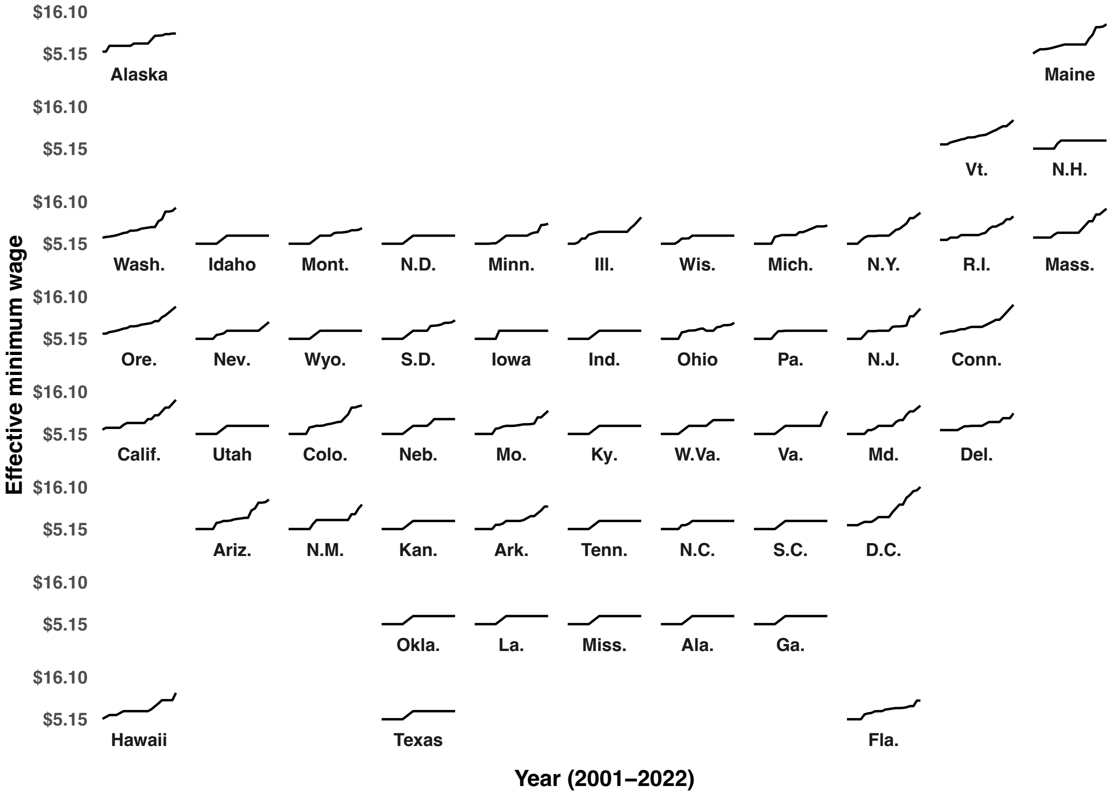

eFigure 1. Effective minimum wages for each state from 2001 to 2022.

Notes: We used the nominal minimum wage on January 1 of each year, based on data compiled by the U.S. Department of Labor.

© 2024 Kavanagh NM et al. *JAMA Network Open.*

eAppendix 3. Coding of Outcomes

Our outcomes comprised 15 measures of mental well-being. All were dichotomized. All were measured contemporaneously and coded to capture respondents who reported having the condition at the time of the survey (or within the designated recall period).

Several questions from the NSCH asked about diagnoses. All were coded to capture children who had both received a diagnosis and still had the condition at the time of the survey (coded as "1"). Any child who previously or never had the condition was coded as "0". This approach allowed children's mental health to improve or "recover" due to changes in household financial status. Since this approach is strict, we also examined the rates of children with "moderate" or "severe" symptoms for each condition, as compared to children with "mild" or no symptoms/condition. These analyses tested whether changes in the minimum wage reduced the proportion of children with especially burdensome diseases and are reported with the sensitivity analyses in **eAppendix 8** of **Supplement 1**.

Below, we have provided the exact question wording (per the NSCH and YRBSS documentation) and coding of all outcomes (**eTable 2**). Any respondents missing a given outcome were dropped pairwise from those analyses.

© 2024 Kavanagh NM et al. *JAMA Network Open.*

eTable 2. Question wording and coding of all mental health outcomes.

Survey	Outcome	Question wording	Coding
**NSCH**<br>**(All children)**	**Currently has depression**	***Variable name: K2Q32A***<br>"Has a doctor or other health care provider EVER told you that this child has?...Depression?"<br>1 = Yes<br>2 = No<br><br>***Variable name: K2Q32B***<br>"If yes, does this child CURRENTLY have the condition?"<br>1 = Yes<br>2 = No<br>*Skip logic: Skip if K2Q32A=2*	Coded such that "1" indicated a child who had received a depression diagnosis <u>and</u> "currently" had the condition at the time of the survey. Any child who previously or never had depression was coded as "0".<br><br>Specifically:<br>1 if K2Q32B = 1<br><br>Otherwise,<br>0 if K2Q32B = 2 or<br>if K2Q32A = 1 or 2
**NSCH**<br>**(All children)**	**Currently has moderate or severe depression**	*Same criteria as "currently has depression."*<br>*In addition:*<br><br>***Variable name: K2Q32C***<br>"If yes, is it:"<br>1 = Mild<br>2 = Moderate<br>3 = Severe<br>*Skip logic: Skip if K2Q32B in (2,.L)*	Coded such that "1" indicated a child who had received a depression diagnosis <u>and</u> had "moderate" or "severe" symptoms at the time of the survey. Any child with mild depression, previous (but not current) depression, or never depression was coded as "0".<br><br>Specifically,<br>1 if K2Q32C = 2 or 3<br><br>Otherwise,<br>0 if K2Q32C = 1 or<br>if K2Q32B = 1 or 2, or<br>if K2Q32A = 1 or 2

Survey	Outcome	Question wording	Coding
**NSCH**<br>**(All children)**	**Currently has anxiety**	***Variable name: K2Q33A***<br>"Has a doctor or other health care provider EVER told you that this child has?...Anxiety Problems?"<br>1 = Yes<br>2 = No<br><br>***Variable name: K2Q33B***<br>"If yes, does this child CURRENTLY have the condition?"<br>1 = Yes<br>2 = No<br>*Skip logic: Skip if K2Q33A=2*	Same logic as "currently has depression."<br><br>1 if K2Q33B = 1<br><br>Otherwise,<br>0 if K2Q33B = 2 or<br>if K2Q33A = 1 or 2
**NSCH**<br>**(All children)**	**Currently has moderate or severe anxiety**	*Same criteria as "currently has anxiety."*<br>*In addition:*<br><br>***Variable name: K2Q33C***<br>"If yes, is it:"<br>1 = Mild<br>2 = Moderate<br>3 = Severe<br>*Skip logic: Skip if K2Q33B in (2,.L)*	Same logic as "currently has moderate or severe depression."<br><br>Specifically,<br>1 if K2Q33C = 2 or 3<br><br>Otherwise,<br>0 if K2Q33C = 1 or<br>if K2Q33B = 1 or 2, or<br>if K2Q33A = 1 or 2

Survey	Outcome	Question wording	Coding
**NSCH**<br>**(All children)**	**Currently has ADD/ADHD**	***Variable name: K2Q31A***<br>"Has a doctor or other health care provider EVER told you that this child has?...Attention Deficit Disorder or Attention-Deficit/Hyperactivity Disorder, that is, ADD or ADHD?"<br>1 = Yes<br>2 = No<br><br>***Variable name: K2Q31B***<br>"If yes, does this child CURRENTLY have the condition?"<br>1 = Yes<br>2 = No<br>*Skip logic: Skip if K2Q31A=2*	Same logic as "currently has depression."<br><br>1 if K2Q31B = 1<br><br>Otherwise,<br>0 if K2Q31B = 2 or<br>if K2Q31A = 1 or 2
**NSCH**<br>**(All children)**	**Currently has moderate or severe ADD/ADHD**	*Same criteria as "currently has ADD/ADHD."*<br>*In addition:*<br><br>***Variable name: K2Q31C***<br>"If yes, is it:"<br>1 = Mild<br>2 = Moderate<br>3 = Severe<br>*Skip logic: Skip if K2Q31B in (2,.L)*	Same logic as "currently has moderate or severe depression."<br><br>Specifically,<br>1 if K2Q31C = 2 or 3<br><br>Otherwise,<br>0 if K2Q31C = 1 or<br>if K2Q31B = 1 or 2, or<br>if K2Q31A = 1 or 2

Survey	Outcome	Question wording	Coding
**NSCH**<br>**(All children)**	**Currently has behavior problems**	***Variable name: K2Q34A***<br>"Has a doctor, other health care provider, or educator EVER told you that this child has?...Behavioral or Conduct Problems?...Examples of educators are teachers and school nurses."<br>1 = Yes<br>2 = No<br><br>***Variable name: K2Q34B***<br>"If yes, does this child CURRENTLY have the condition?"<br>1 = Yes<br>2 = No<br>*Skip logic: Skip if K2Q34A=2*	Same logic as "currently has depression."<br><br>1 if K2Q34B = 1<br><br>Otherwise,<br>0 if K2Q34B = 2 or<br>if K2Q34A = 1 or 2
**NSHC**<br>**(All children)**	**Currently has moderate or severe behavior problems**	*Same criteria as "currently has behavior problems."*<br>*In addition:*<br><br>***Variable name: K2Q34C***<br>"If yes, is it:"<br>1 = Mild<br>2 = Moderate<br>3 = Severe<br>*Skip logic: Skip if K2Q34B in (2,.L)*	Same logic as "currently has moderate or severe depression."<br><br>Specifically,<br>1 if K2Q34C = 2 or 3<br><br>Otherwise,<br>0 if K2Q34C = 1 or<br>if K2Q34B = 1 or 2, or<br>if K2Q34A = 1 or 2

Survey	Outcome	Question wording	Coding
**NSCH (All children)**	**Chronic digestive Issues in past year**	***Variable name: STOMACH*** “DURING THE PAST 12 MONTHS, has this child had FREQUENT or CHRONIC difficulty with any of the following?...Digesting food, including stomach/intestinal problems, constipation, or diarrhea” 1 = Yes 2 = No	1 if STOMACH = 1 Otherwise, 0 if STOMACH = 2
**NSCH (All children)**	**Any unmet health care in past year**	***Variable name: K4Q27*** “DURING THE PAST 12 MONTHS, was there any time when this child needed health care but it was not received?...Health care includes medical care, dental care, vision care, and mental health services.” 1 = Yes 2 = No	1 if K4Q27 = 1 Otherwise, 0 if K4Q27 = 2

Survey	Outcome	Question wording	Coding
**NSCH**<br>**(All children)**	**Any unmet mental health care in past year**	***Variable name: K4Q27***<br>“DURING THE PAST 12 MONTHS, was there any time when this child needed health care but it was not received?...Health care includes medical care, dental care, vision care, and mental health services.”<br>1 = Yes<br>2 = No<br><br>***Variable name: K4Q28X04***<br>“Which types of care was/were not received?...Mental Health Services”<br>1 = selected<br>2 = not selected<br>*Skip logic: Skip if K4Q27=2*	1 if K4Q28X04 = 1<br><br>Otherwise,<br>0 if K4Q27 = 1 or 2
**NSCH**<br>**(All children)**	**7+ school absences In past year**	***Variable name: K7Q02R_R***<br>“DURING THE PAST 12 MONTHS, about how many days did this child miss school because of an illness or injury? Include days missed from any formal home schooling.”<br>1 = No missed school days<br>2 = 1 - 3 days<br>3 = 4 - 6 days<br>4 = 7 - 10 days<br>5 = 11 or more days<br>6 = This child was not enrolled in school<br>*Skip logic: If FORMTYPE in (‘T2’,‘T3’), i.e. ages 6–17*	1 if K7Q02R_R = 4–5<br><br>Otherwise,<br>0 if K7Q02R_R = 1–3, 6

Survey	Outcome	Question wording	Coding
**NSCH**<br>**(All children)**	**Paid employment**<br>**In past year**	***Variable name: K7Q38***<br>"DURING THE PAST 12 MONTHS, did this child participate in:...Any paid work including regular jobs as well as babysitting, cutting grass, or other occasional work?"<br>1 = Yes<br>2 = No<br>*Skip logic: If FORMTYPE in ('T2','T3'), i.e. ages 6–17*	1 if K7Q38 = 1<br>Otherwise,<br>0 if K7Q48 = 2
**YRBSS**<br>**(Adolescents)**	**Sad or hopeless**<br>**In past year**	***Variable name: Q25***<br>"During the past 12 months, did you ever feel so sad or hopeless almost every day for two weeks or more in a row that you stopped doing some usual activities?"<br>A. Yes<br>B. No	1 if Q25 = A (1)<br>Otherwise,<br>0 if Q25 = B (2)
**YRBSS**<br>**(Adolescents)**	**Considered suicide**<br>**In past year**	***Variable name: Q26***<br>"During the past 12 months, did you ever seriously consider attempting suicide?"<br>A. Yes<br>B. No	1 if Q26 = A (1)<br>Otherwise,<br>0 if Q26 = B (2)

Survey	Outcome	Question wording	Coding
**YRBSS (Adolescents)**	**Attempted suicide In past year**	***Variable name: Q28***<br>"During the past 12 months, how many times did you actually attempt suicide?"<br>A. 0 times<br>B. 1 time<br>C. 2 or 3 times<br>D. 4 or 5 times<br>E. 6 or more times	1 if Q28 = B–E (2–5)<br><br>Otherwise,<br>0 if Q28 = A (1)
**YRBSS (Adolescents)**	**Alcohol use in past month**	***Variable name: Q41***<br>"During the past 30 days, on how many days did you have at least one drink of alcohol?"<br>A. 0 days<br>B. 1 or 2 days<br>C. 3 to 5 days<br>D. 6 to 9 days<br>E. 10 to 19 days<br>F. 20 to 29 days<br>G. All 30 days	1 if Q41 = B–G (2–7)<br><br>Otherwise,<br>0 if Q41 = A (1)
**YRBSS (Adolescents)**	**Marijuana use in past month**	***Variable name: Q47***<br>"During the past 30 days, how many times did you use marijuana?"<br>A. 0 times<br>B. 1 or 2 times<br>C. 3 to 9 times<br>D. 10 to 19 times<br>E. 20 to 39 times<br>F. 40 or more times	1 if Q47 = B–F (2–6)<br><br>Otherwise,<br>0 if Q47 = 1

Survey	Outcome	Question wording	Coding
**YRBSS (Adolescents)**	**Physical fight in past year**	***Variable name: Q16***<br>"During the past 12 months, how many times were you in a physical fight?"<br>A. 0 times<br>B. 1 time<br>C. 2 or 3 times<br>D. 4 or 5 times<br>E. 6 or 7 times<br>F. 8 or 9 times<br>G. 10 or 11 times<br>H. 12 or more times	1 If Q16 = B–H (2–8)<br><br>Otherwise,<br>0 If Q16 = A (1)<br><br>**Note:** The YRBSS asked two questions about physical fights; the other specifically asked about fights on school property (Q17). However, many states asked only one question each year. We chose the more inclusive outcome, i.e. any fight, although the results were similarly null and/or conflicting when we examined fights on school property.

eAppendix 4. Individual-Level and State Policy Controls

All fully adjusted models included a variety of individual- and state-level controls (**eTable 3**). Note that the YRBSS has fewer available covariates than the NSCH. These control variables were informed by several previous studies on the minimum wage and children's health,[1–4] plus evidence on inequities in mental health by demographics and socioeconomic status.[5] Importantly, we excluded any individual- or policy-level controls that might be on the causal pathway from rising wages to mental health, including household income, unemployment rates, poverty rates, and the like. Including these potential mediators as covariates might bias our estimates.[6] Indeed, we evaluated one such measure (employment) as an outcome in our analyses.

The state policy controls, in particular, were drawn from two papers by Wehby and colleagues.[3,4] They cover several competing state-level policies that varied over time and could have affected the financial well-being of low-income families. These data were collected from numerous sources, which we document below alongside any coding decisions we made for these controls. The raw and cleaned data tables are available with the paper's replication materials.

Medicaid income eligibility limits were sourced from the Kaiser Family Foundation (https://www.kff.org/statedata/collection/trends-in-medicaid-income-eligibility-limits/). Data for 2001 and 2007 were unavailable, so we used the income limits from October 2000 and July 2006, respectively. For 2009, we used the limits from January, not December, in keeping with the other years. Tennessee had no upper limit for some years, so we used a value of 400% FPL.

Earned income tax credit (EITC) policies were based on data from the Tax Policy Center, run by the Urban Institute and Brookings Institution (https://www.taxpolicycenter.org/statistics/state-eitc-percentage-federal-eitc). For the presence of a state EITC, we coded any state with a non-zero ratio of state-to-federal EITC as 1 and all other states as 0. For states with multiple rates, we used the most generous benefit for which a household with children might be eligible. For example, Wisconsin had three rates corresponding to the number of dependent children for many years, so we used the highest rate. Similarly, if a state offered different refundable and non-refundable rates, we used the higher rate. Any state without an EITC was assigned a rate of 0. The Tax Policy Center was missing data for 2011, so we used the rates from 2010. When coding whether a state's EITC was refundable, any EITC that was at least "partially" refundable was coded as 1; all other states (including those without an EITC) were coded as 0.

Temporary Assistance for Needy Families (TANF) benefits were adapted from the Urban Institute's Welfare Rules Database (https://wrd.urban.org/wrd/Query/query.cfm). We triangulated the maximum benefits (in nominal dollars) for a family of 3 on January 1 of each year by

using the database and a summary table compiled through 2020 (called “Table L5”). If the database was missing a given date, we used the most recent amount or looked to the summary table for guidance. For example, the database was missing values for Colorado from 2001 to 2007; since the summary table reported the same value in both 1996 and 2004 ($365), we used $365 for 2001–2007. In cases of uncertainty or disagreement between the database and the summary table, we documented our decisions in the cleaned data table in the replication package.

eTable 3. Control variables included in minimum wage models.

Level	NSCH models	YRBSS models
**Fixed effects**	State	State
	Year	Year
	Birth cohort*	Birth cohort*
**Individual**	Child’s age	Adolescent’s age
	Child’s sex	Adolescent’s sex
	Child’s race/ethnicity	Adolescent’s race/ethnicity
	Family structure	Adolescent’s grade in high school
	Highest education of any adult in household	
	Household nativity	
**State**	Medicaid income limits for ages 1–5**	Medicaid income limits for ages 1–5**
	Medicaid income limits for ages 6–18**	Medicaid income limits for ages 6–18**
	Presence of state EITC	Presence of state EITC
	State EITC as percent of federal EITC**	State EITC as percent of federal EITC**
	State EITC refundability	State EITC refundability
	Maximum TANF benefit for family of 3**	Maximum TANF benefit for family of 3**

Notes:

The fixed effects were included in all models (labeled “FE only”); the individual- and state-level controls were included only in fully adjusted models (labeled “fully adjusted”).

*The birth cohort fixed effects were constructed by subtracting respondents’ ages from the survey year. They account for distinct generational experiences during the 22-year study period.

**Treated as continuous variables. All other variables were treated as categorical.

eAppendix 5. Corrections for Multiple Comparisons

The risk of type I errors, or false positives due to chance, increases with the number of statistical tests. Given that we examined 15 outcomes, many subgroups, and several modeling approaches, the risk of false positives in our study was considerably greater than 5%.

To reduce this risk, we implemented Bonferroni corrections for multiple hypothesis testing on all analyses in the study. This procedure caps the family-wise error rate for a given analysis at 5% without assuming independence across outcomes. The Bonferroni correction is conservative, resulting in a true family-wise error rate likely lower than 5%,[7] but it allows us to rule out the largest possible associations between the minimum wage and each mental health outcome.

For all TWFE and lifetime minimum wage analyses, which examined 15 outcomes, we used an alpha of 0.05/15 = 0.0033, or a 99.7% CI. This corresponded to a critical value of 2.94. For the difference-in-differences and event study models, which examined only 6 outcomes, we used an alpha of 0.05/6 = 0.0083, or a 99.2% CI and a corresponding critical value of 2.64. The uncorrected and Bonferroni-corrected CIs are provided in most **eFigures** and **eTables**.

eAppendix 6. Approach #1: Two-Way Fixed Effects (TWFE) Models

The standard TWFE analyses are primarily described in the main text. For completeness, we have provided their estimating equations, all regression coefficients for the main models (**eTables 4–5**), and Bonferroni-corrected CIs (**eFigures 2–3**) here in **Supplement 1**. The Bonferroni-corrected CIs were moderately wider than the uncorrected CIs. Even so, we could continue to rule out meaningfully large associations between the minimum wage and all outcomes.

The standard TWFE analyses were specified as follows:

$$Y_{istc} = \beta_1(min.wage)_{st} + \boldsymbol{\beta X_{istc}} + \boldsymbol{\beta Z_{st}} + \Delta_s + \tau_t + \gamma_c + \varepsilon_{istc}$$

where Y_{istc} is the mental health outcome for individual *i* in birth cohort *c* in state *s* in year *t*; $(min.\ wage)_{st}$ is a continuous measure of effective minimum wage in nominal $USD in a given state-year; X_{istc} is a vector of individual-level controls (which varied by survey; see **eTable 3**); Z_{st} is a vector of time-variant state-level policies (**eTable 3**); Δ_s is the time-invariant state fixed effect; τ_t is the survey year fixed effect; γ_c is the birth cohort fixed effect; and ε_{istc} is the error.

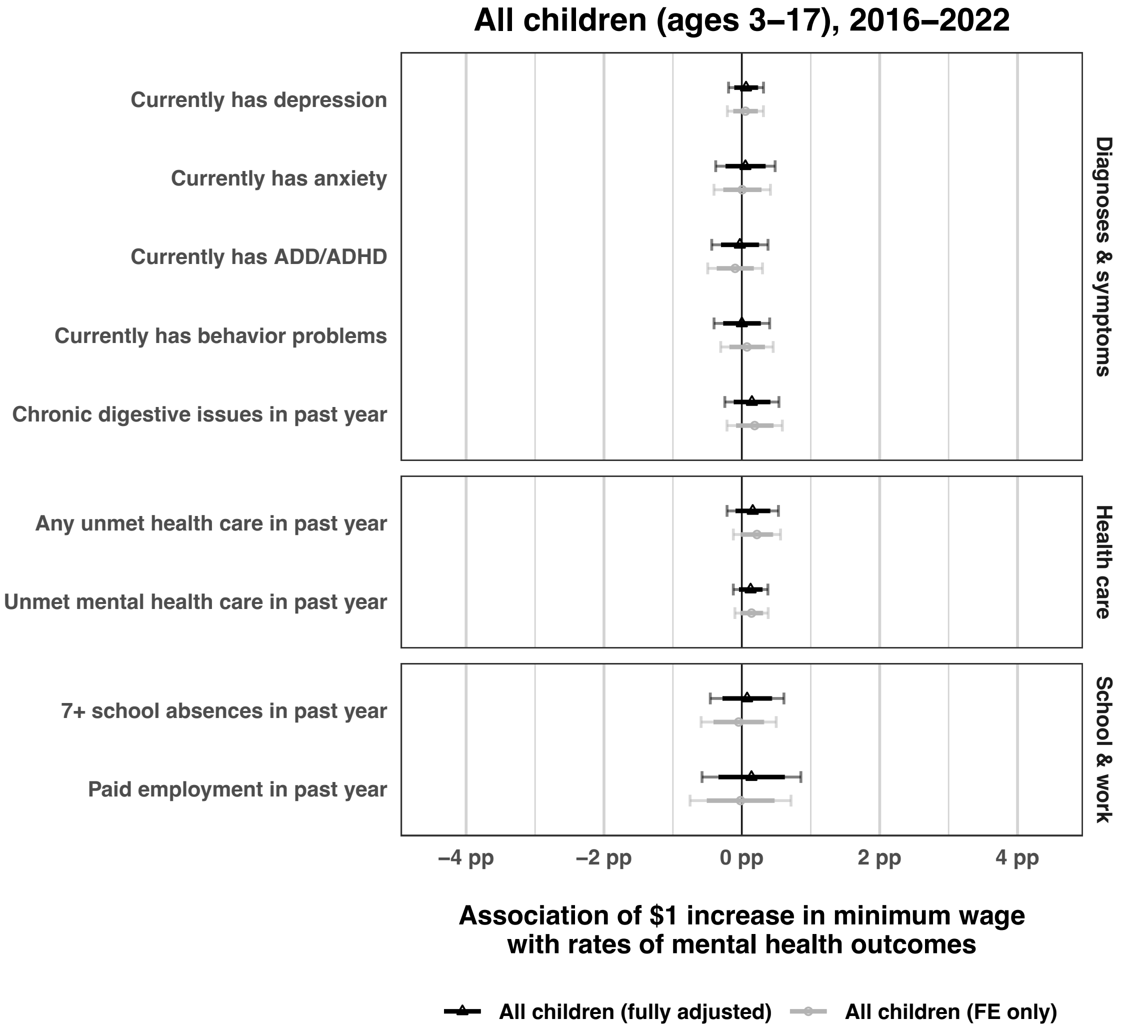

eFigure 2. Standard TWFE models with Bonferroni corrections in the NSCH.

Notes: Re-estimations of the standard OLS TWFE models with Bonferroni corrections for 15 outcomes. The thick lines provide the uncorrected 95% CIs (i.e. with critical values of 1.96), while the thin ones provide the Bonferroni-corrected 99.7% CIs (i.e. with critical values of 2.94). All models included state, year, and birth cohort fixed effects; fully adjusted models also included individual- and state-level controls per **eTable 3**. SEs were clustered at the state level. N= 185,433–238,826. The full regression results (i.e. numeric values) are provided in **eTable 4**.

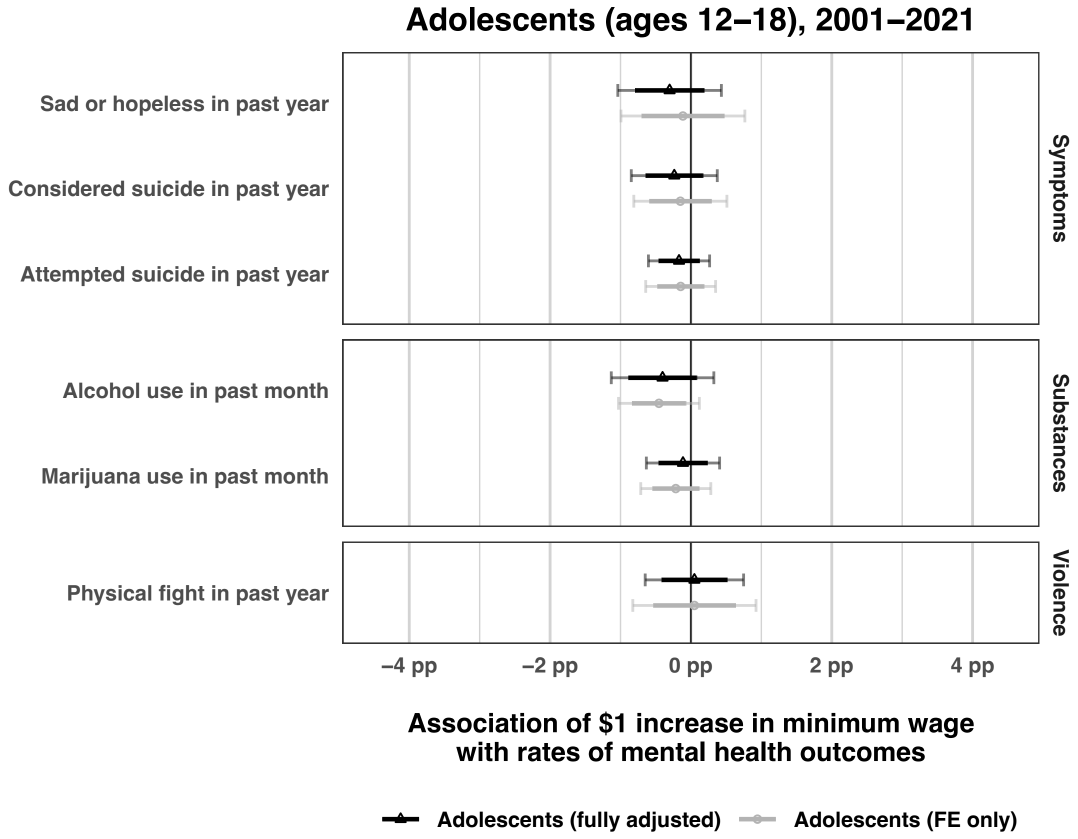

eFigure 3. Standard TWFE models with Bonferroni corrections in the YRBSS.

Notes: Re-estimations of the standard OLS TWFE models with Bonferroni corrections for 15 outcomes. The thick lines provide the uncorrected 95% CIs (i.e. with critical values of 1.96), while the thin ones provide the Bonferroni-corrected 99.7% CIs (i.e. with critical values of 2.94). All models included state, year, and birth cohort fixed effects; fully adjusted models also included individual- and state-level controls per **eTable 3**. SEs were clustered at the state level. N= 1,099,239–1,417,389. The full regression results (i.e. numeric values) are provided in **eTable 5**.

eTable 4. Full regression results for the standard TWFE models in the NSCH.

	Currently has depression		Currently has anxiety		Currently has ADD/ADHD		Currently has behavior problems		Chronic digestive issues in past year		Any unmet health care in past year		Unmet mental health care in past year		7+ school absences in past year		Paid employment in past year	
	FE only	Fully adj.	FE only	Fully adj.	FE only	Fully adj.	FE only	Fully adj.	FE only	Fully adj.	FE only	Fully adj.	FE only	Fully adj.	FE only	Fully adj.	FE only	Fully adj.
**$1 increase in minimum wage**	0.0005	0.0006	0.0001	0.0005	-0.0010	-0.0003	0.0007	0.0000	0.0019	0.0015	0.0022	0.0016	0.0014	0.0013	-0.0005	0.0008	-0.0002	0.0014
	(0.0009)	(0.0009)	(0.0014)	(0.0015)	(0.0014)	(0.0014)	(0.0013)	(0.0014)	(0.0014)	(0.0013)	(0.0012)	(0.0013)	(0.0008)	(0.0009)	(0.0019)	(0.0018)	(0.0025)	(0.0024)
	P=0.564	P=0.484	P=0.969	P=0.724	P=0.475	P=0.838	P=0.568	P=0.998	P=0.179	P=0.279	P=0.065	P=0.218	P=0.092	P=0.142	P=0.805	P=0.675	P=0.939	P=0.570
**4 years old**		-0.0106		-0.0113		-0.0012		0.0123		-0.0081		0.0052		-0.0023				
Reference: 3 years old (except for absences		(0.0016)		(0.0035)		(0.0029)		(0.0046)		(0.0038)		(0.0045)		(0.0015)				
& employment, whose ref. is 6 years old)		P=0.000		P=0.002		P=0.670		P=0.010		P=0.041		P=0.251		P=0.127				
**5 years old**		-0.0208		-0.0151		0.0078		0.0276		-0.0134		0.0061		-0.0010				
Reference: 3 years old (except for absences		(0.0016)		(0.0025)		(0.0037)		(0.0044)		(0.0041)		(0.0036)		(0.0016)				
& employment, whose ref. is 6 years old)		P=0.000		P=0.000		P=0.039		P=0.000		P=0.002		P=0.098		P=0.558				
**6 years old**		-0.0228		-0.0125		0.0150		0.0414		-0.0111		0.0081		0.0050				
Reference: 3 years old (except for absences		(0.0041)		(0.0033)		(0.0041)		(0.0036)		(0.0077)		(0.0045)		(0.0033)				
& employment, whose ref. is 6 years old)		P=0.000		P=0.000		P=0.001		P=0.000		P=0.154		P=0.080		P=0.145				
**7 years old**		-0.0305		-0.0155		0.0308		0.0388		-0.0013		0.0046		0.0002		-0.0079		-0.0497
Reference: 3 years old (except for absences		(0.0029)		(0.0037)		(0.0046)		(0.0048)		(0.0053)		(0.0032)		(0.0033)		(0.0073)		(0.0034)
& employment, whose ref. is 6 years old)		P=0.000		P=0.000		P=0.000		P=0.000		P=0.803		P=0.159		P=0.952		P=0.287		P=0.000
**8 years old**		-0.0390		-0.0121		0.0421		0.0583		-0.0060		0.0080		0.0063		-0.0098		-0.0810
Reference: 3 years old (except for absences		(0.0028)		(0.0037)		(0.0042)		(0.0052)		(0.0087)		(0.0078)		(0.0044)		(0.0059)		(0.0089)
& employment, whose ref. is 6 years old)		P=0.000		P=0.002		P=0.000		P=0.000		P=0.493		P=0.307		P=0.162		P=0.105		P=0.000
**9 years old**		-0.0395		-0.0130		0.0606		0.0608		-0.0051		-0.0024		-0.0006		-0.0090		-0.1224
Reference: 3 years old (except for absences		(0.0023)		(0.0052)		(0.0056)		(0.0040)		(0.0060)		(0.0039)		(0.0024)		(0.0071)		(0.0090)
& employment, whose ref. is 6 years old)		P=0.000		P=0.015		P=0.000		P=0.000		P=0.397		P=0.530		P=0.797		P=0.213		P=0.000
**10 years old**		-0.0479		-0.0175		0.0571		0.0535		0.0001		0.0056		0.0013		-0.0076		-0.1527
Reference: 3 years old (except for absences		(0.0042)		(0.0055)		(0.0068)		(0.0057)		(0.0057)		(0.0069)		(0.0030)		(0.0057)		(0.0096)
& employment, whose ref. is 6 years old)		P=0.000		P=0.003		P=0.000		P=0.000		P=0.983		P=0.422		P=0.671		P=0.192		P=0.000
**11 years old**		-0.0473		-0.0194		0.0472		0.0427		-0.0159		-0.0010		-0.0016		-0.0184		-0.1756
Reference: 3 years old (except for absences		(0.0033)		(0.0047)		(0.0056)		(0.0041)		(0.0083)		(0.0043)		(0.0014)		(0.0078)		(0.0088)
& employment, whose ref. is 6 years old)		P=0.000		P=0.000		P=0.000		P=0.000		P=0.062		P=0.812		P=0.250		P=0.022		P=0.000
**12 years old**		-0.0428		-0.0211		0.0481		0.0428		-0.0107		-0.0040		0.0003		-0.0108		-0.1648
Reference: 3 years old (except for absences		(0.0035)		(0.0047)		(0.0078)		(0.0046)		(0.0054)		(0.0036)		(0.0020)		(0.0090)		(0.0119)
& employment, whose ref. is 6 years old)		P=0.000		P=0.000		P=0.000		P=0.000		P=0.052		P=0.268		P=0.902		P=0.234		P=0.000
**13 years old**		-0.0372		-0.0284		0.0370		0.0323		-0.0089		-0.0068		-0.0047		-0.0104		-0.1782
Reference: 3 years old (except for absences		(0.0043)		(0.0048)		(0.0055)		(0.0041)		(0.0084)		(0.0044)		(0.0015)		(0.0080)		(0.0095)
& employment, whose ref. is 6 years old)		P=0.000		P=0.000		P=0.000		P=0.000		P=0.296		P=0.130		P=0.003		P=0.199		P=0.000
**14 years old**		-0.0312		-0.0140		0.0322		0.0262		-0.0209		-0.0073		-0.0012		-0.0072		-0.1718
Reference: 3 years old (except for absences		(0.0046)		(0.0046)		(0.0075)		(0.0046)		(0.0064)		(0.0041)		(0.0021)		(0.0090)		(0.0102)
& employment, whose ref. is 6 years old)		P=0.000		P=0.004		P=0.000		P=0.000		P=0.002		P=0.079		P=0.591		P=0.427		P=0.000
**15 years old**		-0.0220		-0.0157		0.0210		0.0160		-0.0118		0.0004		-0.0018		-0.0109		-0.1342
Reference: 3 years old (except for absences		(0.0048)		(0.0077)		(0.0052)		(0.0072)		(0.0060)		(0.0032)		(0.0025)		(0.0072)		(0.0113)
& employment, whose ref. is 6 years old)		P=0.000		P=0.047		P=0.000		P=0.030		P=0.053		P=0.892		P=0.466		P=0.140		P=0.000
**16 years old**		-0.0110		-0.0057		0.0098		0.0110		-0.0112		-0.0013		0.0044		-0.0005		-0.0585
Reference: 3 years old (except for absences		(0.0042)		(0.0063)		(0.0049)		(0.0054)		(0.0061)		(0.0044)		(0.0034)		(0.0083)		(0.0127)
& employment, whose ref. is 6 years old)		P=0.011		P=0.370		P=0.054		P=0.045		P=0.070		P=0.777		P=0.202		P=0.956		P=0.000
**17 years old***		N/A		N/A		N/A		N/A		N/A		N/A		N/A		N/A		N/A
Reference: 3 years old (except for absences																		
& employment, whose ref. is 6 years old)																		
**Female**		0.0131		0.0138		-0.0625		-0.0505		0.0073		0.0003		-0.0001		0.0002		-0.0187
Reference: Male		(0.0019)		(0.0027)		(0.0029)		(0.0033)		(0.0025)		(0.0020)		(0.0011)		(0.0024)		(0.0047)
		P=0.000		P=0.000		P=0.000		P=0.000		P=0.005		P=0.889		P=0.932		P=0.942		P=0.000
**Asian, Native Hawaiian, or Pacific Islander**		-0.0277		-0.0489		-0.0186		-0.0286		-0.0283		-0.0191		-0.0049		-0.0391		-0.0458
Reference: American Indian or Alaska Native		(0.0093)		(0.0121)		(0.0147)		(0.0126)		(0.0141)		(0.0082)		(0.0058)		(0.0136)		(0.0275)
		P=0.005		P=0.000		P=0.213		P=0.028		P=0.050		P=0.025		P=0.403		P=0.006		P=0.101
**Black or African Americans**		-0.0249		-0.0519		-0.0039		0.0003		-0.0232		-0.0014		-0.0025		-0.0493		-0.0103
Reference: American Indian or Alaska Native		(0.0098)		(0.0128)		(0.0143)		(0.0129)		(0.0132)		(0.0083)		(0.0060)		(0.0139)		(0.0277)

© 2024 Kavanagh NM et al. *JAMA Network Open.*

	Currently has depression		Currently has anxiety		Currently has ADD/ADHD		Currently has behavior problems		Chronic digestive issues in past year		Any unmet health care in past year		Unmet mental health care in past year		7+ school absences in past year		Paid employment in past year	
	FE only	Fully adj.	FE only	Fully adj.	FE only	Fully adj.	FE only	Fully adj.	FE only	Fully adj.	FE only	Fully adj.	FE only	Fully adj.	FE only	Fully adj.	FE only	Fully adj.
		P=0.014		P=0.000		P=0.789		P=0.979		P=0.086		P=0.863		P=0.680		P=0.001		P=0.713
**Hispanic/Latino**		-0.0176		-0.0162		0.0017		-0.0147		-0.0030		0.0010		-0.0013		-0.0032		0.0062
Reference: American Indian or Alaska Native		(0.0092)		(0.0126)		(0.0140)		(0.0129)		(0.0136)		(0.0090)		(0.0056)		(0.0144)		(0.0285)
		P=0.063		P=0.205		P=0.905		P=0.259		P=0.828		P=0.909		P=0.811		P=0.828		P=0.827
**White**		-0.0142		-0.0016		0.0138		-0.0103		-0.0107		-0.0065		-0.0023		-0.0030		0.0509
Reference: American Indian or Alaska Native		(0.0091)		(0.0124)		(0.0145)		(0.0126)		(0.0135)		(0.0078)		(0.0052)		(0.0129)		(0.0265)
		P=0.126		P=0.899		P=0.346		P=0.418		P=0.430		P=0.414		P=0.657		P=0.819		P=0.061
**Other or mixed race**		-0.0077		0.0028		0.0217		0.0078		-0.0172		0.0047		0.0048		0.0058		0.0176
Reference: American Indian or Alaska Native		(0.0101)		(0.0129)		(0.0142)		(0.0126)		(0.0142)		(0.0095)		(0.0063)		(0.0137)		(0.0282)
		P=0.449		P=0.827		P=0.132		P=0.542		P=0.230		P=0.625		P=0.448		P=0.674		P=0.536
**Two parents, not married**		0.0198		0.0205		0.0227		0.0340		0.0223		0.0198		0.0064		0.0256		-0.0025
Reference: Two parents, married		(0.0028)		(0.0033)		(0.0055)		(0.0041)		(0.0078)		(0.0037)		(0.0029)		(0.0079)		(0.0054)
		P=0.000		P=0.000		P=0.000		P=0.000		P=0.006		P=0.000		P=0.030		P=0.002		P=0.649
**Single parent**		0.0215		0.0276		0.0332		0.0423		0.0193		0.0152		0.0079		0.0412		-0.0334
Reference: Two parents, married		(0.0025)		(0.0041)		(0.0053)		(0.0035)		(0.0043)		(0.0028)		(0.0010)		(0.0042)		(0.0045)
		P=0.000		P=0.000		P=0.000		P=0.000		P=0.000		P=0.000		P=0.000		P=0.000		P=0.000
**Another family structure**		0.0619		0.0830		0.1012		0.1287		0.0176		0.0349		0.0183		-0.0011		-0.0490
Reference: Two parents, married		(0.0099)		(0.0162)		(0.0150)		(0.0238)		(0.0088)		(0.0054)		(0.0057)		(0.0188)		(0.0140)
		P=0.000		P=0.000		P=0.000		P=0.000		P=0.050		P=0.000		P=0.002		P=0.953		P=0.001
**Family structure not provided**		0.0145		0.0329		0.0241		0.0448		0.0113		0.0148		0.0107		-0.0257		-0.0439
Reference: Two parents, married		(0.0058)		(0.0083)		(0.0111)		(0.0134)		(0.0145)		(0.0059)		(0.0066)		(0.0136)		(0.0105)
		P=0.016		P=0.000		P=0.035		P=0.002		P=0.438		P=0.016		P=0.111		P=0.065		P=0.000
**High school (including vocational)**		0.0019		0.0163		0.0129		0.0035		0.0096		-0.0126		-0.0005		0.0147		0.0145
Reference: Less than high school		(0.0026)		(0.0071)		(0.0072)		(0.0058)		(0.0065)		(0.0042)		(0.0020)		(0.0084)		(0.0094)
		P=0.451		P=0.026		P=0.077		P=0.550		P=0.149		P=0.005		P=0.798		P=0.085		P=0.127
**Some college or associate degree**		0.0090		0.0256		0.0169		0.0089		0.0185		-0.0040		0.0059		0.0219		0.0338
Reference: Less than high school		(0.0035)		(0.0065)		(0.0082)		(0.0059)		(0.0087)		(0.0038)		(0.0019)		(0.0095)		(0.0084)
		P=0.013		P=0.000		P=0.044		P=0.133		P=0.038		P=0.306		P=0.003		P=0.026		P=0.000
**College degree or higher**		0.0003		0.0252		0.0031		-0.0076		0.0065		-0.0182		0.0020		-0.0069		0.0592
Reference: Less than high school		(0.0023)		(0.0065)		(0.0062)		(0.0050)		(0.0082)		(0.0051)		(0.0019)		(0.0083)		(0.0072)
		P=0.911		P=0.000		P=0.615		P=0.133		P=0.435		P=0.001		P=0.318		P=0.411		P=0.000
**Adult education not provided**		-0.0022		0.0095		0.0176		0.0090		-0.0027		-0.0037		-0.0020		0.0158		0.0648
Reference: Less than high school		(0.0079)		(0.0114)		(0.0179)		(0.0136)		(0.0182)		(0.0130)		(0.0058)		(0.0195)		(0.0269)
		P=0.785		P=0.409		P=0.330		P=0.510		P=0.883		P=0.776		P=0.729		P=0.421		P=0.020
**Second-generation household**		0.0142		0.0174		0.0157		0.0121		0.0046		-0.0186		0.0018		0.0103		0.0545
Reference: First-generation household		(0.0031)		(0.0053)		(0.0101)		(0.0031)		(0.0090)		(0.0086)		(0.0023)		(0.0095)		(0.0058)
		P=0.000		P=0.002		P=0.127		P=0.000		P=0.608		P=0.034		P=0.427		P=0.282		P=0.000
**Third-generation household or higher**		0.0290		0.0501		0.0620		0.0396		0.0092		-0.0171		0.0051		0.0396		0.1073
Reference: First-generation household		(0.0030)		(0.0062)		(0.0125)		(0.0050)		(0.0083)		(0.0069)		(0.0018)		(0.0087)		(0.0055)
		P=0.000		P=0.000		P=0.000		P=0.000		P=0.270		P=0.016		P=0.006		P=0.000		P=0.000
**Nativity not provided**		0.0127		0.0038		0.0270		-0.0081		0.0032		-0.0245		0.0016		0.0584		0.0971
Reference: First-generation household		(0.0048)		(0.0078)		(0.0156)		(0.0119)		(0.0103)		(0.0082)		(0.0052)		(0.0158)		(0.0120)
		P=0.011		P=0.626		P=0.090		P=0.501		P=0.760		P=0.005		P=0.760		P=0.001		P=0.000
**Medicare income eligibility limit, ages 1–5**		0.3648		0.0477		-0.1542		-0.3476		-0.4946		-0.4399		-0.1913		-0.6680		0.0582
**(% of federal poverty level)**		(0.1293)		(0.1015)		(0.3865)		(0.2656)		(0.2473)		(0.3949)		(0.2356)		(0.1327)		(0.2700)
		P=0.007		P=0.640		P=0.692		P=0.197		P=0.051		P=0.271		P=0.421		P=0.000		P=0.830
**Medicare income eligibility limit, ages 6-18**		-0.2489		-0.0339		0.0863		0.2497		0.3466		0.2743		0.1240		0.4087		-0.0480
**(% of federal poverty level)**		(0.0825)		(0.0706)		(0.2465)		(0.1808)		(0.1601)		(0.2499)		(0.1507)		(0.0887)		(0.1781)
		P=0.004		P=0.633		P=0.728		P=0.173		P=0.035		P=0.278		P=0.414		P=0.000		P=0.789
**State has EITC (yes/no)**		-0.0048		-0.0237		-0.0072		-0.0089		0.0048		-0.0195		-0.0047		0.0021		0.0128
		(0.0061)		(0.0093)		(0.0052)		(0.0057)		(0.0053)		(0.0109)		(0.0039)		(0.0150)		(0.0108)
		P=0.436		P=0.014		P=0.169		P=0.123		P=0.375		P=0.080		P=0.233		P=0.888		P=0.238
**State EITC as percent of federal (%)**		0.0190		0.0178		0.0235		-0.0045		0.0039		0.0106		0.0039		0.0320		0.0271
		(0.0109)		(0.0131)		(0.0101)		(0.0083)		(0.0099)		(0.0077)		(0.0039)		(0.0204)		(0.0143)
		P=0.089		P=0.180		P=0.025		P=0.588		P=0.699		P=0.174		P=0.322		P=0.123		P=0.064
**State EITC is refundable (yes/no)**		0.0063		0.0136		0.0153		0.0024		-0.0017		0.0044		-0.0018		-0.0136		-0.0340

	Currently has depression		Currently has anxiety		Currently has ADD/ADHD		Currently has behavior problems		Chronic digestive issues in past year		Any unmet health care in past year		Unmet mental health care in past year		7+ school absences in past year		Paid employment in past year	
	FE only	Fully adj.	FE only	Fully adj.	FE only	Fully adj.	FE only	Fully adj.	FE only	Fully adj.	FE only	Fully adj.	FE only	Fully adj.	FE only	Fully adj.	FE only	Fully adj.
		(0.0033)		(0.0049)		(0.0012)		(0.0022)		(0.0027)		(0.0041)		(0.0020)		(0.0145)		(0.0035)
		P=0.062		P=0.007		P=0.000		P=0.271		P=0.520		P=0.294		P=0.393		P=0.353		P=0.000
**Maximum TANF benefit for family of 3 ($)**		0.0000		0.0000		0.0000		0.0000		0.0000		0.0000		0.0000		0.0000		0.0000
		(0.0000)		(0.0000)		(0.0000)		(0.0000)		(0.0000)		(0.0000)		(0.0000)		(0.0000)		(0.0000)
		P=0.588		P=0.762		P=0.815		P=0.557		P=0.196		P=0.107		P=0.223		P=0.835		P=0.502
**State, age, and birth cohort FEs**	Yes	Yes	Yes	Yes	Yes	Yes	Yes	Yes	Yes	Yes	Yes	Yes	Yes	Yes	Yes	Yes	Yes	Yes
**Cluster-robust SEs**	State	State	State	State	State	State	State	State	State	State	State	State	State	State	State	State	State	State
**Number of children**	238,746	238,746	238,643	238,643	237,938	237,938	238,826	238,826	237,822	237,822	238,612	238,612	238,612	238,612	186,905	186,905	185,433	185,433
**Adjusted R2**	0.032	0.044	0.031	0.044	0.024	0.052	0.006	0.035	0.002	0.005	0.005	0.011	0.003	0.006	0.012	0.022	0.225	0.261

Notes: This table provides full regression results for the standard TWFE models in **Figure 1** and **eFigure 2**. Cluster-robust SEs are provided in parentheses. Fixed effects not shown. *Terms were collinear with other controls and/or fixed effects in some models.

eTable 5. Full regression results for the standard TWFE models in the YRBSS.

	Sad or hopeless in past year		Considered suicide in past year		Attempted suicide in past year		Alcohol use in past month		Marijuana use in path month		Physical fight in past year	
	FE only	Fully adj.	FE only	Fully adj.	FE only	Fully adj.	FE only	Fully adj.	FE only	Fully adj.	FE only	Fully adj.
**$1 increase in minimum wage**	-0.0011<br>(0.0030)<br>P=0.706	-0.0030<br>(0.0025)<br>P=0.232	-0.0015<br>(0.0022)<br>P=0.508	-0.0024<br>(0.0021)<br>P=0.262	-0.0015<br>(0.0017)<br>P=0.392	-0.0017<br>(0.0015)<br>P=0.259	-0.0045<br>(0.0020)<br>P=0.025	-0.0040<br>(0.0025)<br>P=0.111	-0.0022<br>(0.0017)<br>P=0.210	-0.0011<br>(0.0018)<br>P=0.527	0.0005<br>(0.0030)<br>P=0.868	0.0005<br>(0.0024)<br>P=0.836
**13 years old***<br>*Reference: 12 years old or younger*		-0.1882<br>(0.0206)<br>P=0.000		N/A		N/A		-0.2389<br>(0.0195)<br>P=0.000		-0.2408<br>(0.0197)<br>P=0.000		-0.2011<br>(0.0270)<br>P=0.000
**14 years old**<br>*Reference: 12 years old or younger*		-0.2275<br>(0.0083)<br>P=0.000		-0.2552<br>(0.0116)<br>P=0.000		-0.2727<br>(0.0120)<br>P=0.000		-0.2569<br>(0.0135)<br>P=0.000		-0.2814<br>(0.0200)<br>P=0.000		-0.2849<br>(0.0145)<br>P=0.000
**15 years old**<br>*Reference: 12 years old or younger*		-0.1201<br>(0.0042)<br>P=0.000		0.0880<br>(0.0159)<br>P=0.000		0.0448<br>(0.0164)<br>P=0.009		-0.1316<br>(0.0066)<br>P=0.000		-0.1518<br>(0.0088)<br>P=0.000		-0.1516<br>(0.0080)<br>P=0.000
**16 years old**<br>*Reference: 12 years old or younger*		-0.0999<br>(0.0047)<br>P=0.000		-0.1213<br>(0.0063)<br>P=0.000		-0.1341<br>(0.0059)<br>P=0.000		-0.1117<br>(0.0066)<br>P=0.000		-0.1198<br>(0.0099)<br>P=0.000		-0.1279<br>(0.0089)<br>P=0.000
**17 years old***<br>*Reference: 12 years old or younger*		N/A		0.2194<br>(0.0156)<br>P=0.000		0.1820<br>(0.0196)<br>P=0.000		N/A		N/A		N/A
**18 years old or older***<br>*Reference: 12 years old or younger*		N/A		N/A		N/A		N/A		N/A		N/A
**Female**<br>*Reference: Male*		0.1688<br>(0.0030)<br>P=0.000		0.0912<br>(0.0023)<br>P=0.000		0.0390<br>(0.0027)<br>P=0.000		0.0258<br>(0.0032)<br>P=0.000		-0.0236<br>(0.0023)<br>P=0.000		-0.1345<br>(0.0049)<br>P=0.000
**Sex not provided**<br>*Reference: Male*		0.2141<br>(0.0215)<br>P=0.000		0.2086<br>(0.0186)<br>P=0.000		0.1966<br>(0.0266)<br>P=0.000		0.1086<br>(0.0238)<br>P=0.000		0.0797<br>(0.0173)<br>P=0.000		0.0681<br>(0.0261)<br>P=0.012
**Asian, Native Hawaiian, or Pacific Islander**<br>*Reference: American Indian or Alaska Native*		-0.0617<br>(0.0102)<br>P=0.000		-0.0460<br>(0.0101)<br>P=0.000		-0.0510<br>(0.0106)<br>P=0.000		-0.1474<br>(0.0145)<br>P=0.000		-0.1657<br>(0.0150)<br>P=0.000		-0.1354<br>(0.0136)<br>P=0.000
**Black or African American**<br>*Reference: American Indian or Alaska Native*		-0.0590<br>(0.0081)<br>P=0.000		-0.0574<br>(0.0094)<br>P=0.000		-0.0341<br>(0.0098)<br>P=0.001		-0.1110<br>(0.0126)<br>P=0.000		-0.0628<br>(0.0172)<br>P=0.001		-0.0043<br>(0.0144)<br>P=0.765
**Hispanic/Latino**<br>*Reference: American Indian or Alaska Native*		-0.0053<br>(0.0092)<br>P=0.570		-0.0328<br>(0.0095)<br>P=0.001		-0.0308<br>(0.0099)<br>P=0.003		-0.0099<br>(0.0120)<br>P=0.414		-0.0684<br>(0.0153)<br>P=0.000		-0.0435<br>(0.0137)<br>P=0.003
**White**<br>*Reference: American Indian or Alaska Native*		-0.0616<br>(0.0079)<br>P=0.000		-0.0383<br>(0.0088)<br>P=0.000		-0.0663<br>(0.0100)<br>P=0.000		0.0127<br>(0.0116)<br>P=0.278		-0.0839<br>(0.0165)<br>P=0.000		-0.1010<br>(0.0136)<br>P=0.000
**Multiple races, non-Hispanic**<br>*Reference: American Indian or Alaska Native*		0.0081<br>(0.0094)<br>P=0.397		0.0185<br>(0.0096)<br>P=0.060		-0.0217<br>(0.0114)<br>P=0.062		-0.0070<br>(0.0109)<br>P=0.527		-0.0404<br>(0.0160)<br>P=0.015		-0.0199<br>(0.0156)<br>P=0.208
**Race/ethnicity not provided**<br>*Reference: American Indian or Alaska Native*		-0.0290<br>(0.0086)<br>P=0.002		-0.0240<br>(0.0098)<br>P=0.018		-0.0011<br>(0.0113)<br>P=0.922		-0.0344<br>(0.0122)<br>P=0.007		-0.0791<br>(0.0159)<br>P=0.000		-0.0312<br>(0.0135)<br>P=0.026
**10th grade**<br>*Reference: 9th grade*		-0.0133<br>(0.0047)<br>P=0.007		-0.0117<br>(0.0026)<br>P=0.000		-0.0206<br>(0.0021)<br>P=0.000		0.0215<br>(0.0032)<br>P=0.000		0.0017<br>(0.0025)<br>P=0.495		-0.0541<br>(0.0035)<br>P=0.000
**11th grade**		-0.0350		-0.0300		-0.0470		0.0471		0.0010		-0.1079

	Sad or hopeless in past year		Considered suicide in past year		Attempted suicide in past year		Alcohol use in past month		Marijuana use in path month		Physical fight in past year	
	FE only	Fully adj.	FE only	Fully adj.	FE only	Fully adj.	FE only	Fully adj.	FE only	Fully adj.	FE only	Fully adj.
Reference: 9th grade		(0.0058)<br>P=0.000		(0.0030)<br>P=0.000		(0.0026)<br>P=0.000		(0.0047)<br>P=0.000		(0.0041)<br>P=0.802		(0.0045)<br>P=0.000
**12th grade**<br>*Reference: 9th grade*		-0.0654<br>(0.0072)<br>P=0.000		-0.0500<br>(0.0034)<br>P=0.000		-0.0686<br>(0.0035)<br>P=0.000		0.0951<br>(0.0072)<br>P=0.000		0.0103<br>(0.0058)<br>P=0.085		-0.1438<br>(0.0059)<br>P=0.000
**Grade not provided**<br>*Reference: 9th grade*		-0.0032<br>(0.0085)<br>P=0.711		0.0142<br>(0.0065)<br>P=0.036		0.0351<br>(0.0081)<br>P=0.000		0.0949<br>(0.0089)<br>P=0.000		0.0615<br>(0.0098)<br>P=0.000		0.0140<br>(0.0106)<br>P=0.193
**Medicare income eligibility limit, age 1-5 (% of federal poverty level)**		-0.0077<br>(0.0136)<br>P=0.577		-0.0249<br>(0.0105)<br>P=0.023		-0.0157<br>(0.0125)<br>P=0.216		0.0112<br>(0.0233)<br>P=0.634		0.0092<br>(0.0104)<br>P=0.382		0.0000<br>(0.0114)<br>P=0.997
**Medicare income eligibility limit, age 6-18 (% of federal poverty level)**		0.0070<br>(0.0115)<br>P=0.546		0.0225<br>(0.0093)<br>P=0.020		0.0172<br>(0.0109)<br>P=0.123		-0.0131<br>(0.0203)<br>P=0.522		-0.0047<br>(0.0094)<br>P=0.622		-0.0015<br>(0.0100)<br>P=0.880
**State has EITC (yes/no)**		-0.0024<br>(0.0126)<br>P=0.852		0.0001<br>(0.0098)<br>P=0.991		-0.0106<br>(0.0086)<br>P=0.225		0.0044<br>(0.0088)<br>P=0.624		0.0094<br>(0.0053)<br>P=0.081		-0.0221<br>(0.0132)<br>P=0.102
**State EITC as percent of federal (%)**		0.0376<br>(0.0142)<br>P=0.011		0.0290<br>(0.0073)<br>P=0.000		0.0208<br>(0.0118)<br>P=0.083		-0.0024<br>(0.0169)<br>P=0.887		-0.0357<br>(0.0086)<br>P=0.000		0.0521<br>(0.0173)<br>P=0.004
**State EITC is refundable (yes/no)**		0.0108<br>(0.0118)<br>P=0.364		-0.0026<br>(0.0092)<br>P=0.777		0.0003<br>(0.0076)<br>P=0.972		-0.0048<br>(0.0075)<br>P=0.527		-0.0068<br>(0.0042)<br>P=0.109		0.0145<br>(0.0105)<br>P=0.176
**Maximum TANF benefit for family of 3 ($)**		0.0000<br>(0.0000)<br>P=0.450		0.0000<br>(0.0000)<br>P=0.259		0.0000<br>(0.0000)<br>P=0.383		0.0000<br>(0.0000)<br>P=0.942		0.0000<br>(0.0000)<br>P=0.970		-0.0001<br>(0.0000)<br>P=0.006
**State, age, and birth cohort FEs**	Yes	Yes	Yes	Yes	Yes	Yes	Yes	Yes	Yes	Yes	Yes	Yes
**Cluster-robust SEs**	State	State	State	State	State	State	State	State	State	State	State	State
**Number of adolescents**	1,417,389	1,417,389	1,306,377	1,306,377	1,099,239	1,099,239	1,337,125	1,337,125	1,402,825	1,402,825	1,107,872	1,107,872
**Adjusted R2**	0.013	0.051	0.006	0.025	0.004	0.021	0.054	0.069	0.016	0.024	0.018	0.057

Notes: This table provides full regression results for the standard TWFE models in **Figure 2** and **eFigure 3**. Cluster-robust SEs are provided in parentheses. Fixed effects not shown. *Terms were collinear with other controls and/or fixed effects in some models.

eAppendix 7. Approach #1: TWFE Subgroup Analyses

We tested the association between the minimum wage and mental health for several populations that were more likely to earn near the minimum wage (and, therefore, more likely to experience an improvement in their health after it was raised) (subgroups listed in **eTable 6**).

For these analyses, we subsetted each dataset to the indicated respondents and re-fitted the main models. This approach is identical to fully interacted TWFE models:

$$Y_{istc} = \beta_1[(min.wage)_{st} * (group)_{istc}] + \beta_2(min.wage)_{st} + \beta_3(group)_{istc} +$$
$$\boldsymbol{\beta X_{istc}} + \boldsymbol{\beta}[\boldsymbol{X_{istc}} * (group)_{istc}] + \boldsymbol{\beta Z_{st}} + \boldsymbol{\beta}[\boldsymbol{Z_{st}} * (group)_{istc}] +$$
$$\Delta_s + \Delta_s * (group)_{istc} + \tau_t + \tau_t * (group)_{istc} + \gamma_c + \gamma_c * (group)_{istc} + \varepsilon_{ist}$$

whereby the minimum wage variable, all controls, and all FEs are also interacted with a binary variable, $group_{istc}$, defined for each population such that inclusion = 0 and non-inclusion = 1. For example, Black and Hispanic/Latino children were coded as 0 and all other children who had complete information on race/ethnicity were coded as 1. In the fully interacted model, β_2 provides the association for the population of interest, e.g. Black and Hispanic/Latino children.

There was no clear, consistent evidence of improvements for any population or any outcome (**eFigures 4–5**). Occasional 95% CIs were statistically significant from zero, but these models (1) did not show a consistent pattern within outcomes, e.g. raising the minimum wage was negatively associated with rates of anxiety for less educated households in the NSCH but no other populations; (2) did not show a consistent direction within populations, e.g. for less educated households, raising the minimum wage was negatively associated with two outcomes, positively associated with one outcome, and null for all other outcomes; and (3) were no longer significant after Bonferroni corrections. As a result, we interpreted these analyses as generally null.

eTable 6. List of subgroup analyses for standard TWFE models.

Survey	Population
**NSCH**	Less than 200 FPL%
**NSCH**	Adults with high school or less<br>*That is, all households for which no adult completed more than high school*
**NSCH**	Black or Hispanic/Latino
**NSCH**	First- or second-generation
**NSCH**	Adolescents, age 13–17
**NSCH**	Not urban or principal city<br>*That is, children not in any metropolitan statistical area (MSA, or denser urban areas of the U.S.) or any of their principal cities*
**YRBSS***	Black or Hispanic/Latino

Notes: *The YRBSS has fewer available demographic and socioeconomic characteristics than the NSCH, so we cannot replicate most subgroup analyses with the YRBSS.

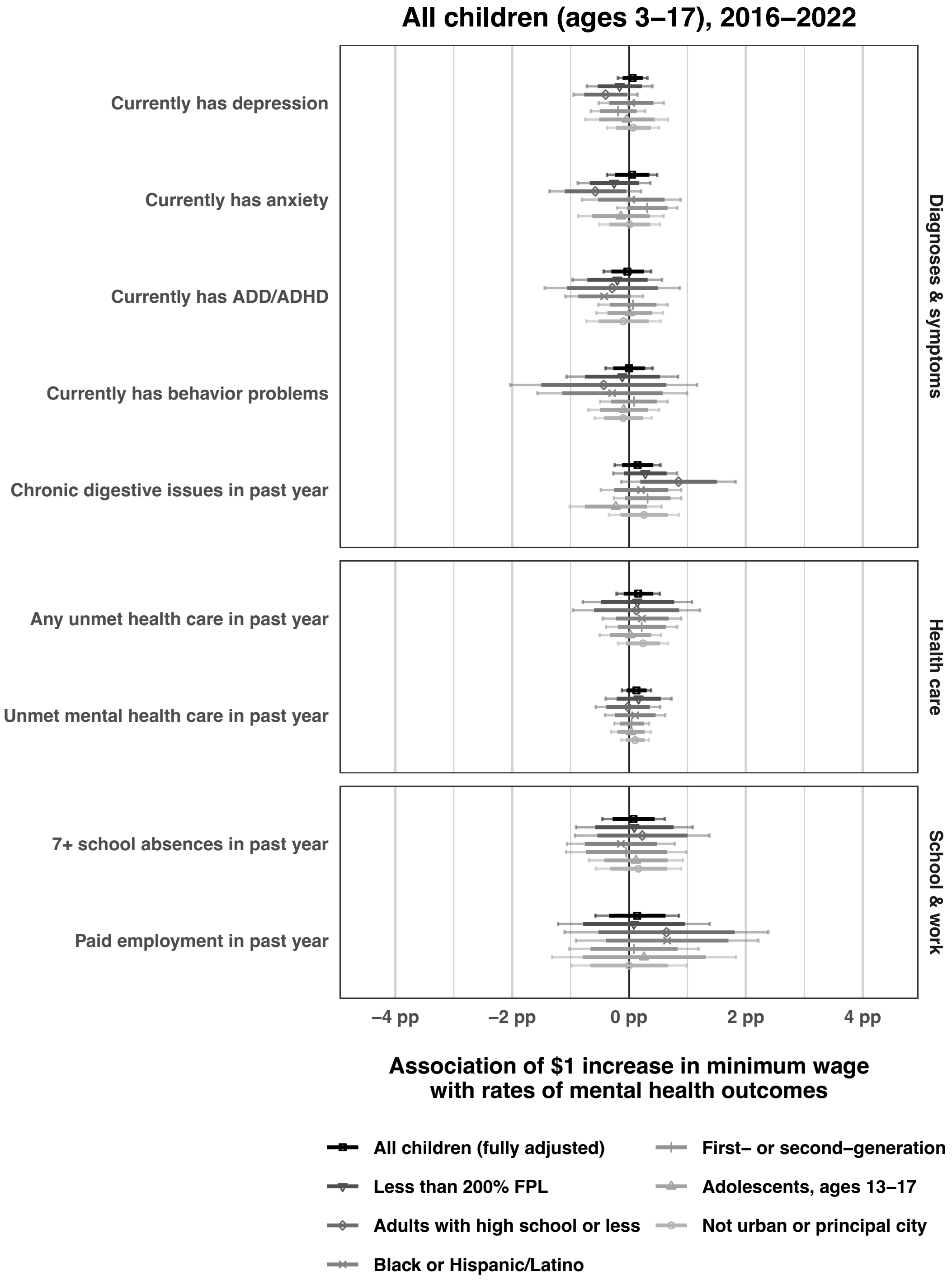

eFigure 4. Subgroup TWFE models in the NSCH.

Notes: Re-estimations of the standard TWFE models within the indicated populations. All models were adjusted for state, year, and birth cohort fixed effects, plus individual- and state-level controls per **eTable 3**. SEs were clustered at the state level. 95% CIs (thick) and 99.7% CIs for Bonferroni corrections (thin) are provided. N=30,043–127,925 for the subgroup models.

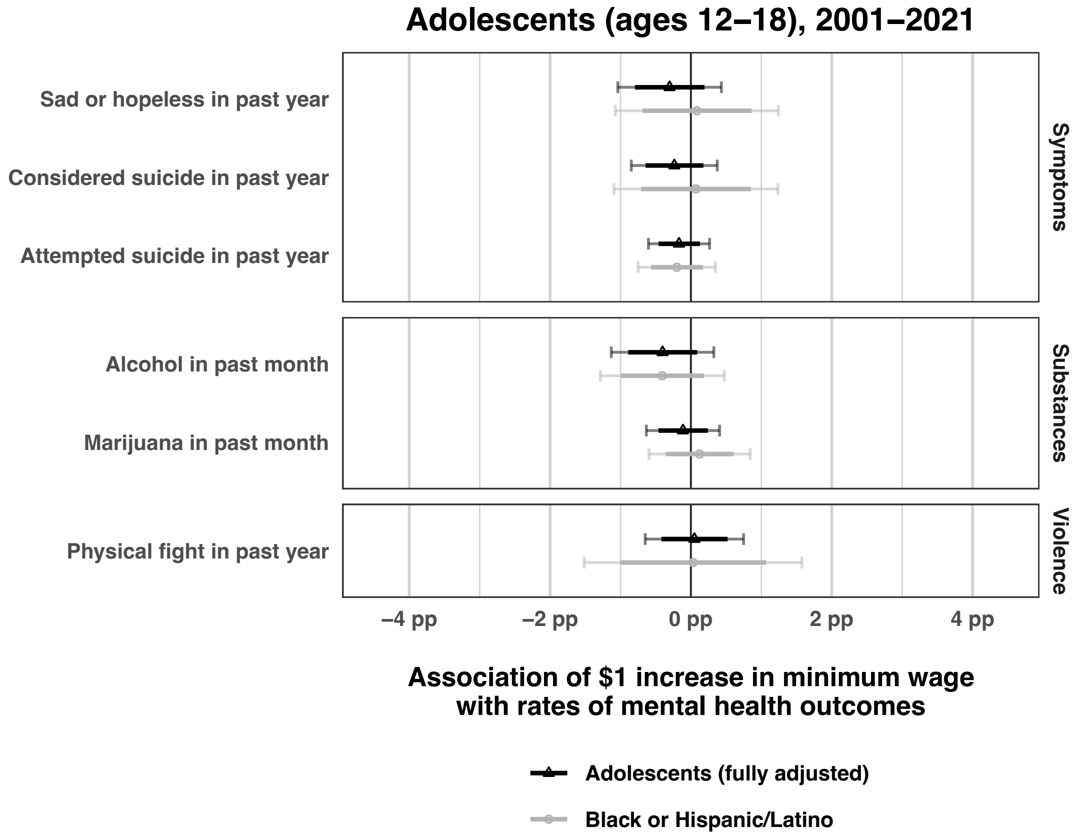

eFigure 5. Subgroup TWFE models in the YRBSS.

Notes: Re-estimations of the standard TWFE models within the indicated populations. All models were adjusted for state, year, and birth cohort fixed effects, plus individual- and state-level controls per **eTable 3**. SEs were clustered at the state level. 95% CIs (thick) and 99.7% CIs for Bonferroni corrections (thin) are provided. N=300,472–424,216 for the subgroup models.

eAppendix 8. Approach #1: TWFE Sensitivity Analyses

We examined the robustness of the standard TWFE results to several sensitivity tests:

(1) <u>Estimations using real wages (i.e. inflation-adjusted in 2020 dollars).</u>

A concern with the standard TWFE models might be that the year fixed effects did not adequately account for rising costs during the study period. As such, we adjusted all minimum wages to 2020 dollars using the Consumer Price Index (CPI) for January each year. The results are substantively identical to the models using nominal wages (**eFigures 6–7**).

(2) <u>Minimum wages lagged by 1 year.</u>

Another concern might be that rises in take-home pay were not fully realized by the time people were surveyed. Relatedly, many outcomes were recalled and might have predated recent changes in minimum wage policies. That is, the NSCH was collected in the second half of each year, and the YRBS was collected in the first half. Meanwhile, we used the minimum wages (and other state-level controls) as of January 1 each year. This means that recalled outcomes, e.g. sadness or hopelessness in the past year, could have predated a policy change.

As such, we lagged the minimum wage policies by one year. For example, a respondent surveyed in 2022 was assigned the minimum wage in their state on January 1, 2021. These lagged models ensured that any policy changes fully pre-dated all recalled outcomes. They provided generally null, otherwise conflicting evidence for the association between minimum wages and mental health (**eFigures 6–7**). A few outcomes in the NSCH retrieved significant positive associations at the 5% level, one in the YRBSS retrieved a negative association, and the remainder were null. None remained statistically significant after the Bonferroni corrections. Taken together, the lagged models did not provide compelling evidence of a beneficial association.

(3) <u>Estimations using binomial logistic regression.</u>

Another concern might be that OLS models improperly captured the functional form of binary outcomes. As such, we estimated binomial logistic regressions in the “survey” package in R (v. 4.2–1). These models estimated the odds ratio for each outcome given a $1 increase in the minimum wage. None provided evidence of significant associations (**eFigures 8–9**).

(4) <u>Estimations using level-log models.</u>

Another functional form concern might be that the OLS models examined changes in absolute wages, not relative wages. A $1 increase may be less meaningful compared to a base rate

of, say, $10 than to $7. As such, we took the natural log of the nominal minimum wage and estimated level-log models using OLS. After re-scaling the coefficients, these models estimated the association between a 10% increase in the minimum wage and the percentage-point prevalence of each outcome. The estimates are virtually identical to the standard TWFE models and do not provide evidence of significant associations in either survey (**eFigures 10–11**).

(5) <u>Alternate standard error structures.</u>

For all main analyses, we clustered the standard errors at the state level. Given that the minimum wage is set by each state, clustering at this level would traditionally be considered appropriate for econometric analyses. However, recent econometric evidence has suggested that typical estimators for cluster-robust standard errors can be overly conservative when a non-negligible fraction of clusters in the population are sampled.[8] Such was the case here since we observed all states (plus Washington, D.C.) in the NSCH models and the vast majority of states in the YRBSS models. As such, the state-clustered errors may have overstated the uncertainty in the association between the minimum wage and children's mental health outcomes.

To reduce this concern, we implemented models with the nested clusters recommended by the NSCH and YRBSS for estimating population prevalences, based on each survey's sampling design (**eFigures 12–13**). For the NSCH, the alternate standard errors nested the survey's sampling strata within each state. For the YRBSS, they nested the survey's sampling strata within each state's primary sampling units. These alternative constructions reflect the distinct sampling designs of the two surveys. In principle, nested errors may inflate the number of clusters relative to the true number of units with varying treatment statuses, thereby understating the uncertainty. Even so, the nested clusters produced substantively similar CIs as the state clusters.

(6) <u>Diagnostic outcomes stratified by symptom severity.</u>

Another concern might be that the diagnostic outcomes in the NSCH were too "strict," as rising minimum wages may have been insufficient to fully relieve children's mental health conditions. However, they may have been sufficient to lessen the symptom burden of these conditions. As such, we tested the associations between nominal minimum wages and the rates of children with "moderate" or "severe" depression, anxiety, ADD/ADHD, and behavior problems, as opposed to "mild" or no such condition. Put differently, these analyses tested whether changes in the minimum wage were associated with reduced rates of children with especially burdensome diseases. However, these models provided no evidence of reductions in symptom burden (**eFigure 14**). The

exact question wording and coding of these outcomes are provided in **eTable 2**. Note that the YRBSS did not provide analogous questions for us to do a similar analysis with that survey.

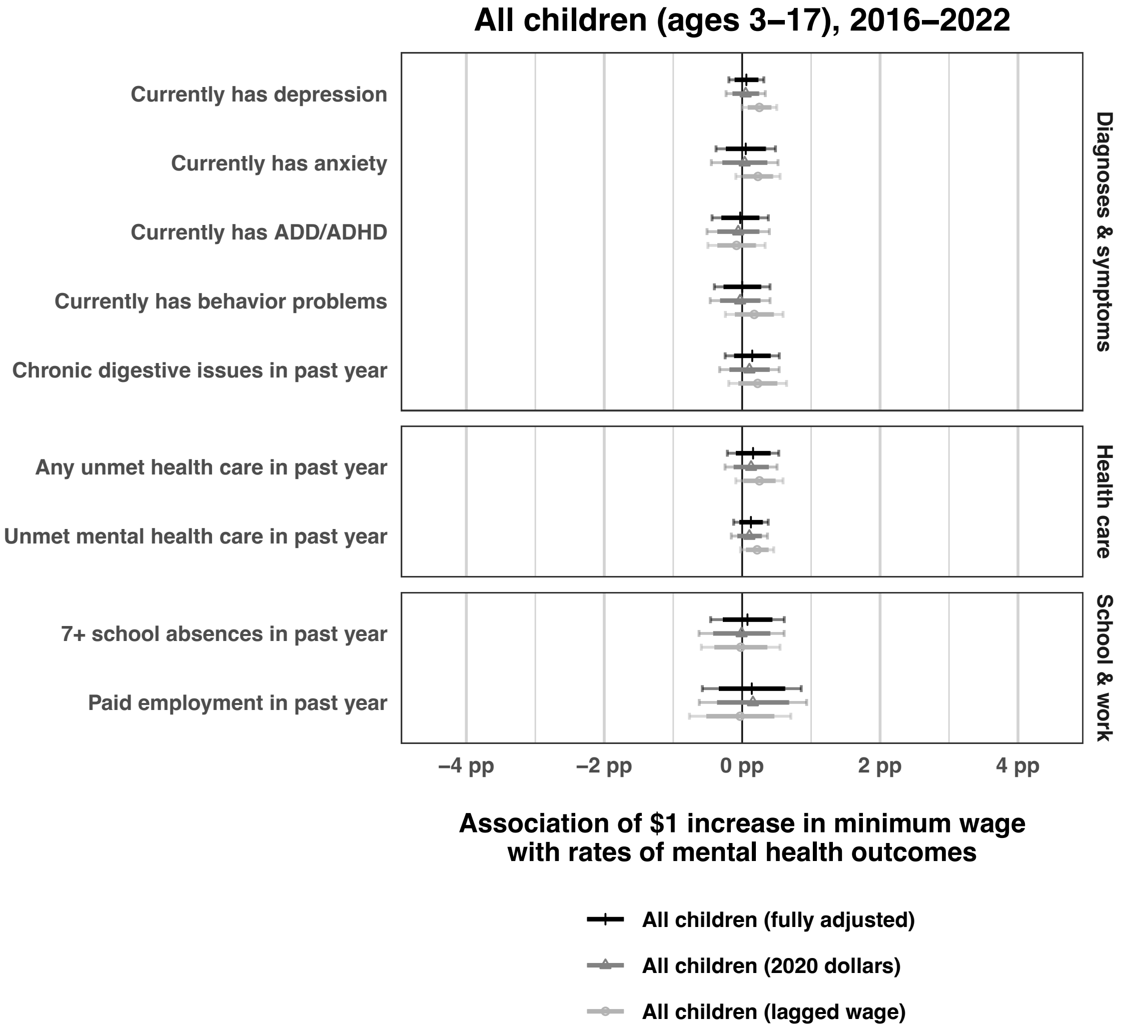

eFigure 6. TWFE models with lagged and inflation-adjusted wages in the NSCH.

Notes: Re-estimations of the standard TWFE models using either the effective minimum wage adjusted for inflation in 2020 dollars or the nominal minimum wage lagged by one year, compared to the standard models. All models were adjusted for individual- and state-level controls per **eTable 3**, plus state, year, and birth cohort fixed effects. SEs were clustered at the state level. 95% CIs (thick) and 99.7% CIs for Bonferroni corrections (thin) are provided. N=185,433–238,826.

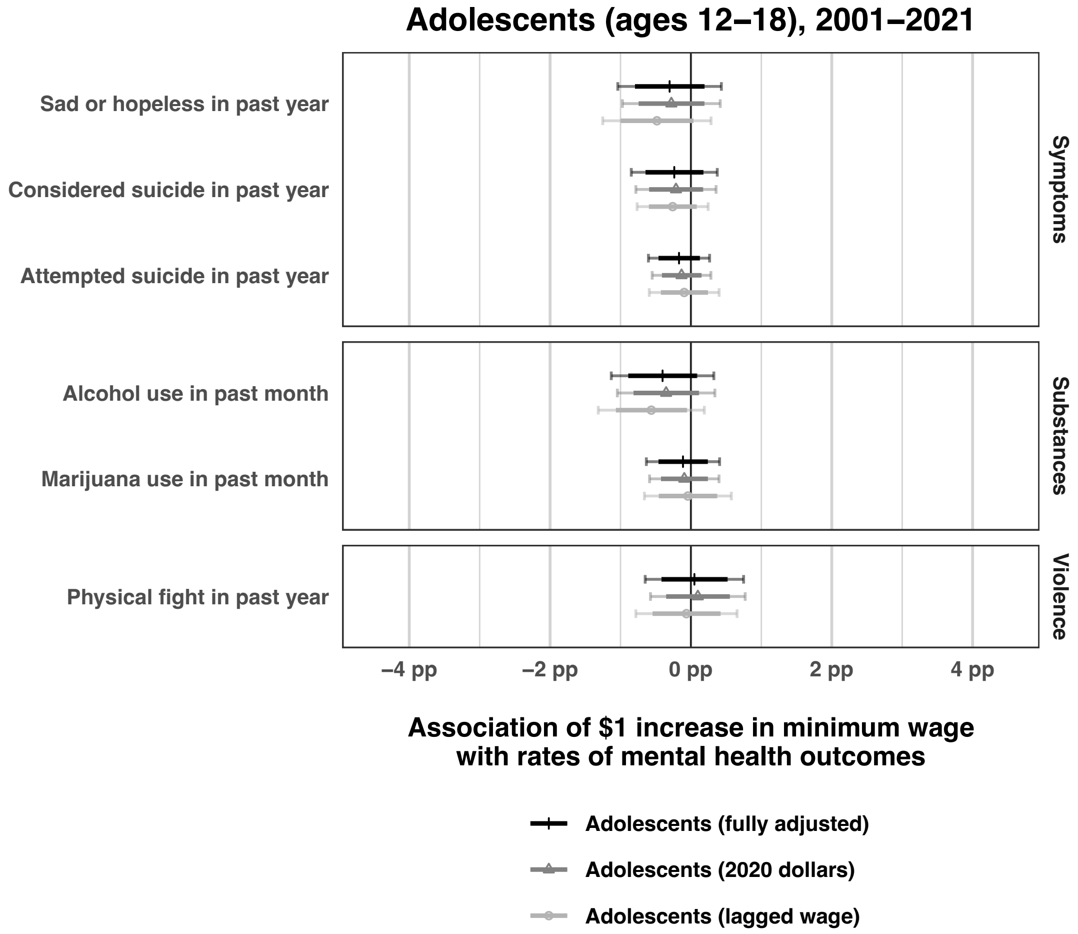

eFigure 7. TWFE models with lagged and inflation-adjusted wages in the YRBSS.

Notes: Re-estimations of the standard TWFE models using either the effective minimum wage adjusted for inflation in 2020 dollars or the nominal minimum wage lagged by one year, compared to the standard models. All models were adjusted for individual- and state-level controls per **eTable 3**, plus state, year, and birth cohort fixed effects. SEs were clustered at the state level. 95% CIs (thick) and 99.7% CIs for Bonferroni corrections (thin) are provided. N=1,099,239–1,417,389.

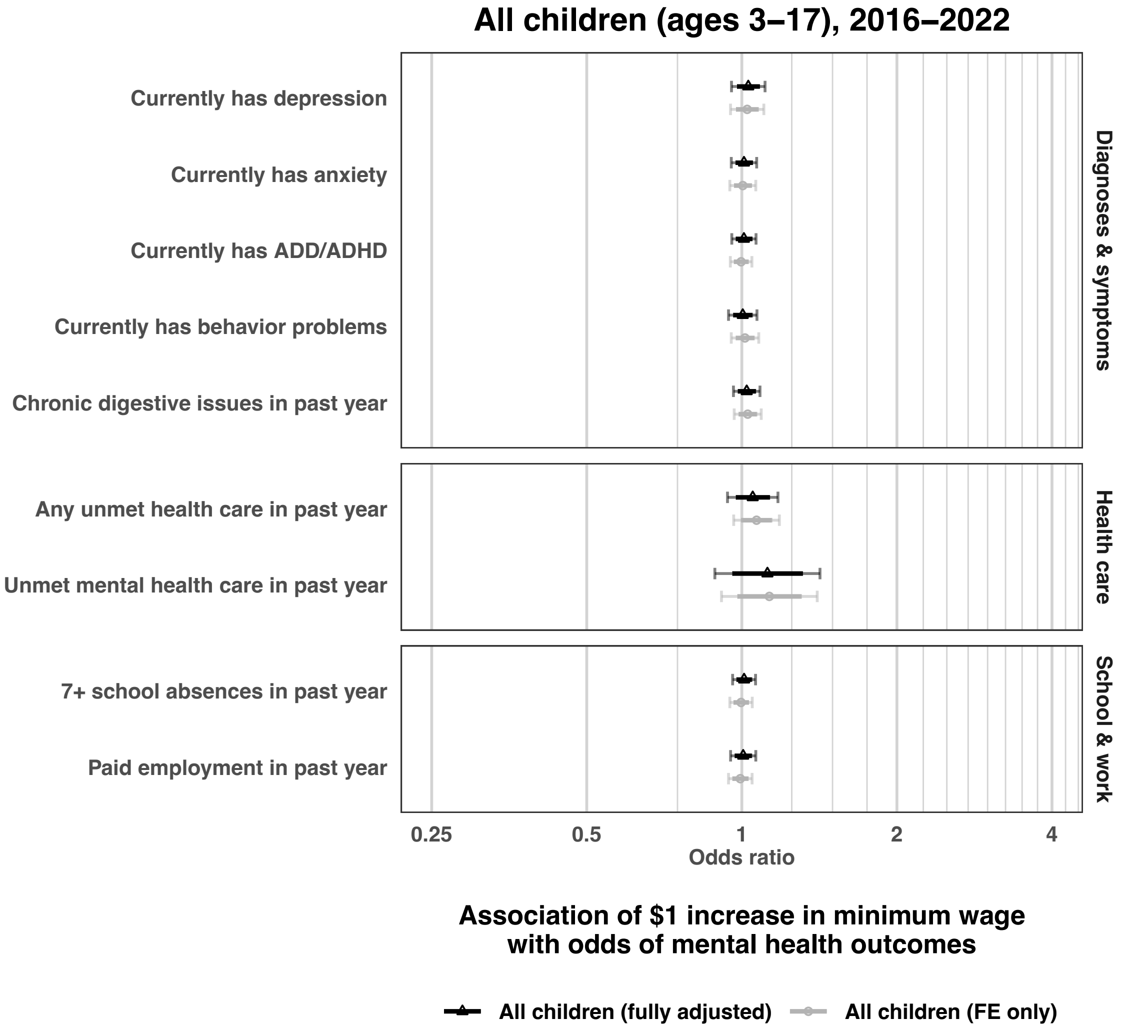

eFigure 8. TWFE models using logistic regression in the NSCH.

Notes: Re-estimations of the standard TWFE models by binomial logistic regression. All models included state, year, and birth cohort fixed effects; fully adjusted models also included individual- and state-level controls per **eTable 3**. SEs were clustered at the state level. 95% CIs (thick) and 99.7% CIs for Bonferroni corrections (thin) are provided. N=185,433–238,826.

© 2024 Kavanagh NM et al. *JAMA Network Open.*

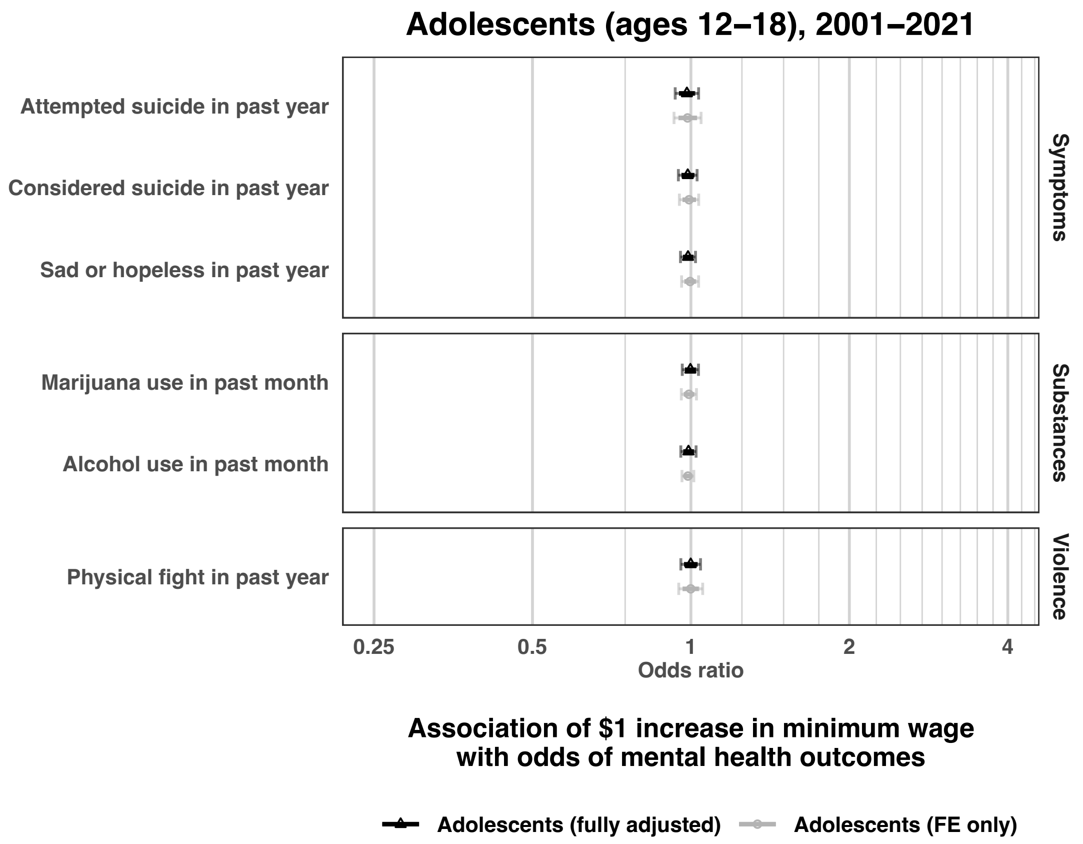

eFigure 9. TWFE models using logistic regression in the YRBSS.

Notes: Re-estimations of the standard TWFE models by binomial logistic regression. All models included state, year, and birth cohort fixed effects; fully adjusted models also included individual- and state-level controls per **eTable 3**. SEs were clustered at the state level. 95% CIs (thick) and 99.7% CIs for Bonferroni corrections (thin) are provided. N=1,099,239–1,417,389.

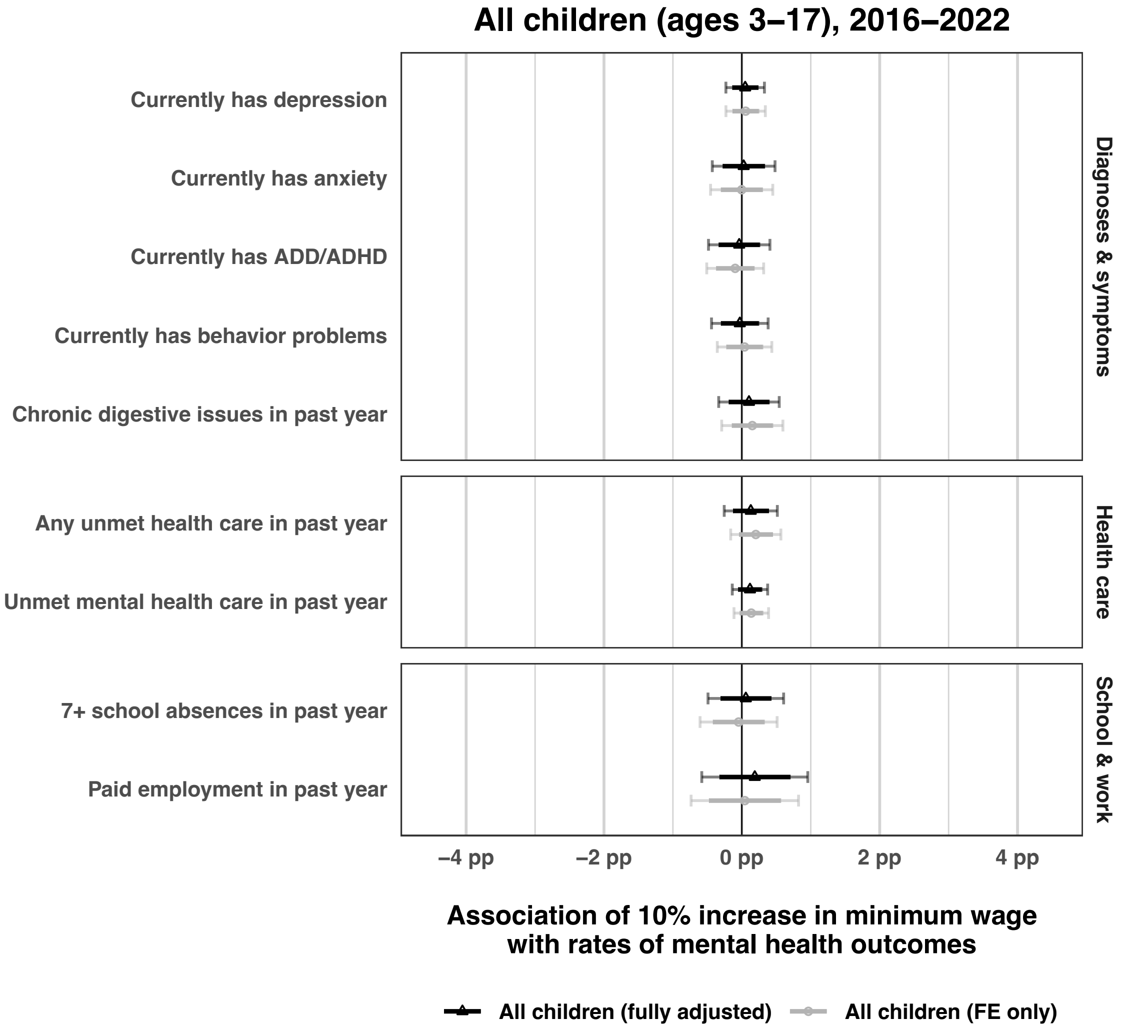

eFigure 10. Level-log TWFE models in the NSCH.

Notes: Re-estimations of the standard TWFE models using the natural log of the nominal minimum wage as the main exposure. The coefficients have been re-scaled to estimate the association between a 10% increase in the minimum wage and the prevalence of each outcome. All models included state, year, and birth cohort fixed effects; fully adjusted models also included individual- and state-level controls per **eTable 3**. SEs were clustered at the state level. 95% CIs (thick) and 99.7% CIs for Bonferroni corrections (thin) are provided. N=185,433–238,826**.**

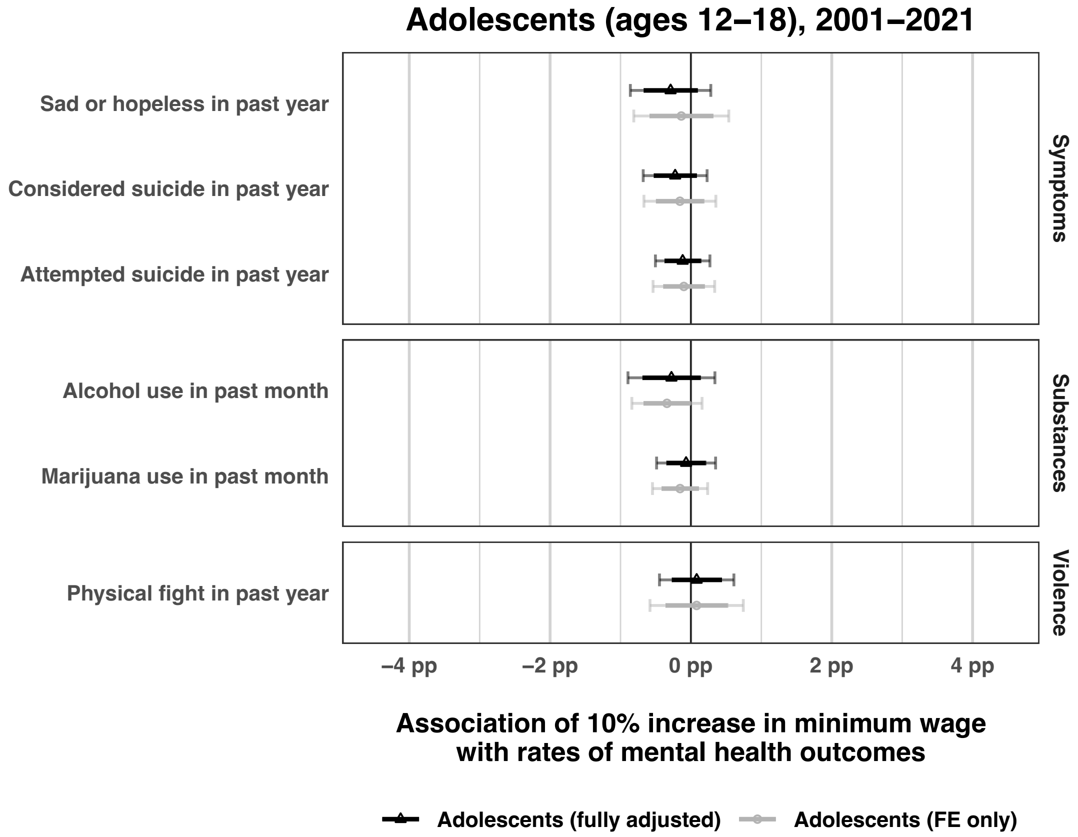

eFigure 11. Level-log TWFE models in the YRBSS.

Notes: Re-estimations of the standard TWFE models using the natural log of the nominal minimum wage as the main exposure. The coefficients have been re-scaled to estimate the association between a 10% increase in the minimum wage and the prevalence of each outcome. All models included state, year, and birth cohort fixed effects; fully adjusted models also included individual- and state-level controls per **eTable 3**. SEs were clustered at the state level. 95% CIs (thick) and 99.7% CIs for Bonferroni corrections (thin) are provided. N=1,099,239–1,417,389**.**

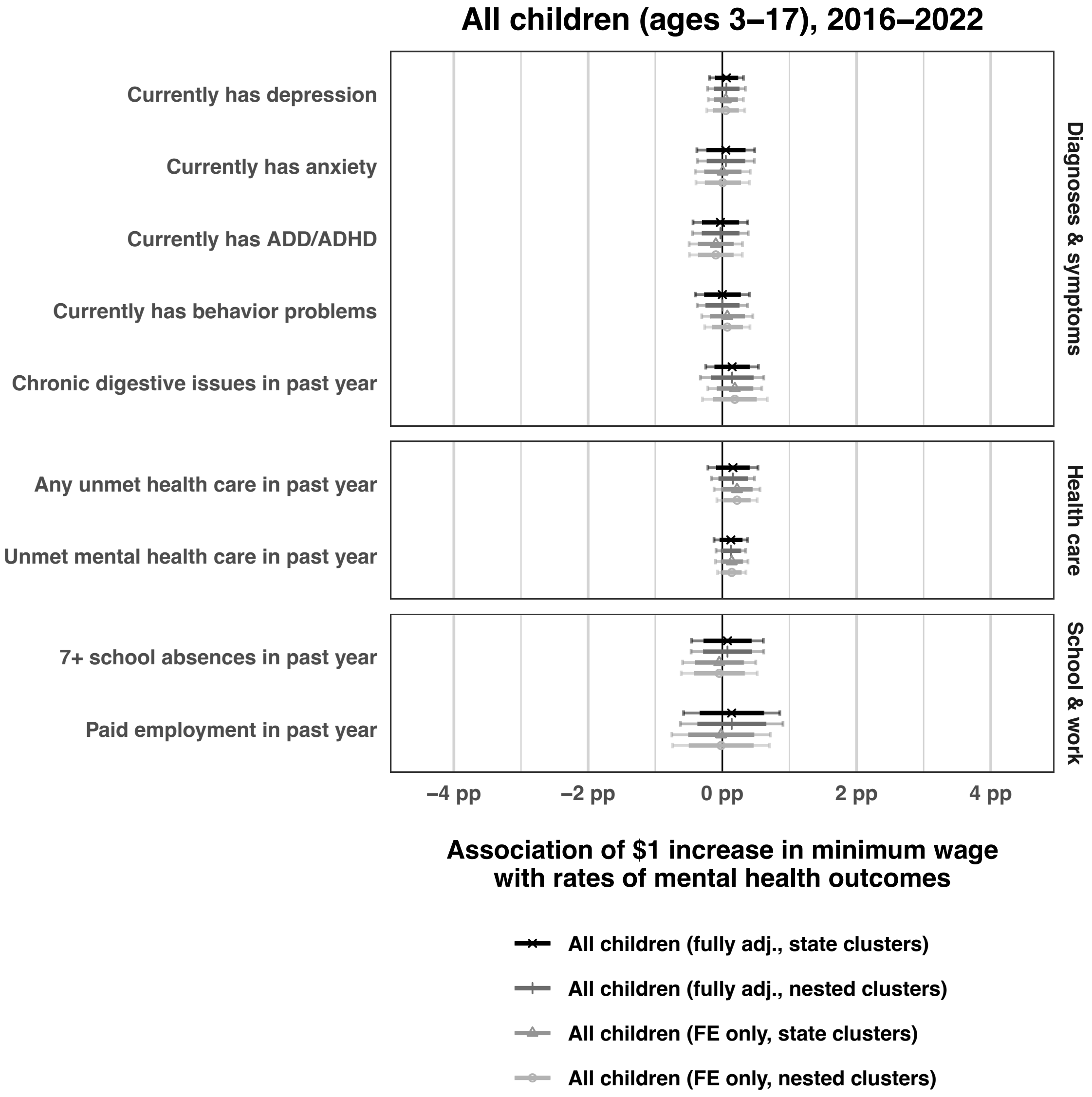

eFigure 12. TWFE models using nested clusters in the NSCH.

Notes: Re-estimations of the standard TWFE models with SEs clustered per the nested survey design, compared to state-clustered models. All models included state, year, and birth cohort FEs; fully adjusted models also included individual- and state-level controls per **eTable 3**. 95% CIs (thick) and 99.7% CIs for Bonferroni corrections (thin) are provided. N=185,433–238,826.

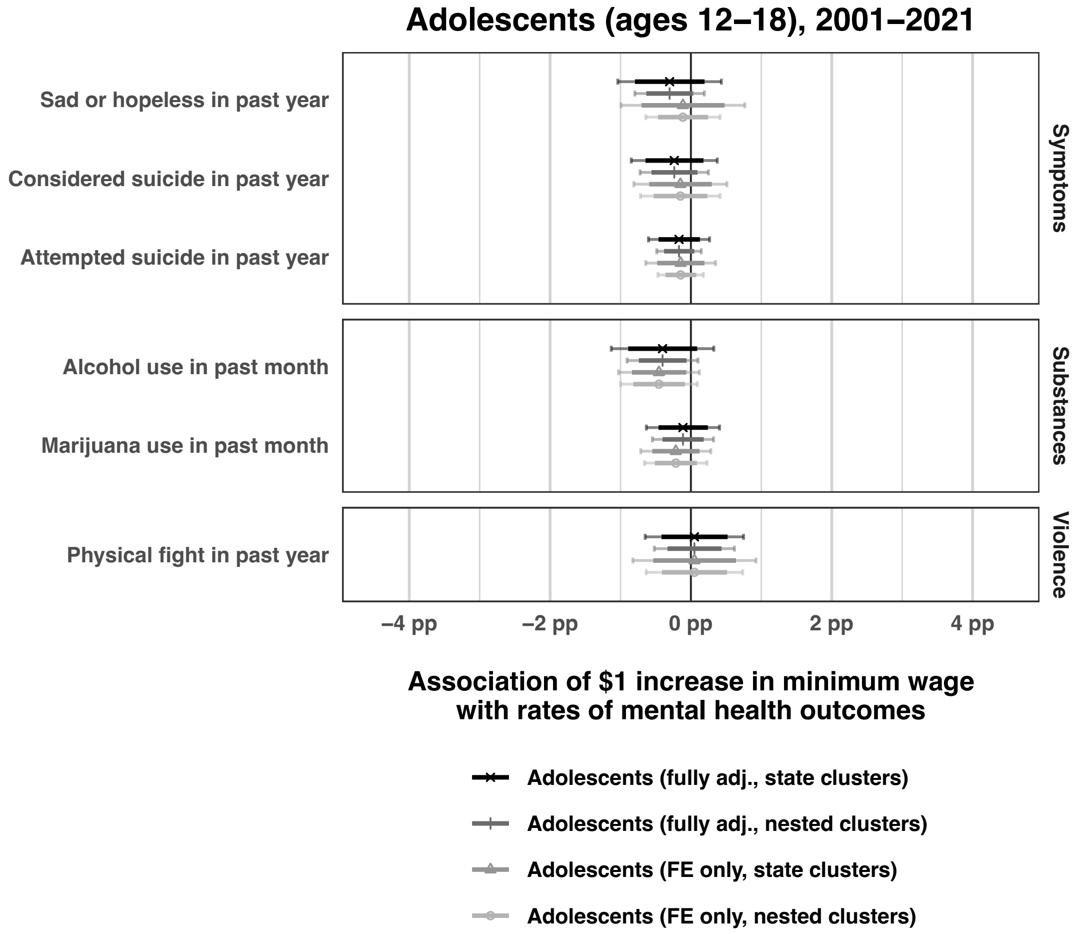

eFigure 13. TWFE models using nested clusters in the YRBSS.

Notes: Re-estimations of the standard TWFE models with SEs clustered per the nested survey design, compared to state-clustered models. All models included state, year, and birth cohort FEs; fully adjusted models also included individual- and state-level controls per **eTable 3**. 95% CIs (thick) and 99.7% CIs for Bonferroni corrections (thin) are provided. N=1,099,239–1,417,389.

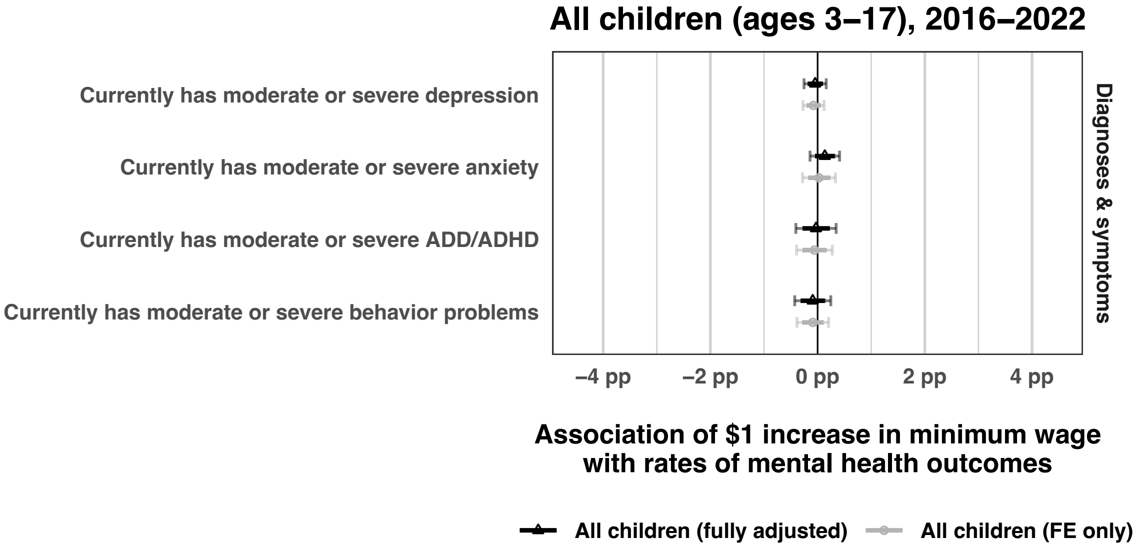

eFigure 14. TWFE models for symptom severity in the NSCH.

Notes: Based on OLS TWFE models that examined the rates of children with “moderate” or “severe” symptoms of the indicated conditions, as opposed to “mild” symptoms or no such condition. All models included state, year, and birth cohort FEs; fully adjusted models also included individual- and state-level controls per **eTable 3**. SEs were clustered at the state level. 95% CIs (thick) and 99.7% CIs for Bonferroni corrections (thin) are provided. N=237,938–238,826.

eAppendix 9. Approach #2: Lifetime Minimum Wage Models

Motivation. A concern about the standard TWFE analyses might be that they modeled contemporaneous changes in mental health and inadequately accounted for longer-term investments. For example, Wehby et al found that children exposed to a higher minimum wage earlier in life had better physical health later in childhood.[3,4] Their work relied on a human capital model, whereby cumulative, long-term investments in well-being were more likely to improve health.

The same could be true for mental health. For example, higher wages might grant families access to structural opportunities, such as higher-quality schools or neighborhoods, that have a lasting impact on their child's mental health. Or perhaps families were able to gradually accumulate wealth that they later tapped into when their child's mental health was threatened.

Methods. As such, we implemented lifetime minimum wage models. These adapted the standard TWFE models with a continuous measure of a child's lifetime minimum wage:

$$Y_{istc} = \beta_1(lifetime\ wage)_{st} + \boldsymbol{\beta X_{istc}} + \boldsymbol{\beta Z_{st}} + \Delta_s + \tau_t + \gamma_c + \varepsilon_{istc}$$

where Y_{istc} is the mental health outcome for individual *i* in birth cohort *c* in state *s* in year *t*; $(lifetime\ wage)_{st}$ is a continuous measure of the average effective minimum wage to which a child was likely exposed throughout their life; X_{istc} is a vector of individual-level controls (see **eTable 3**); Z_{st} is a vector of time-variant state-level policies (**eTable 3**); Δ_s is the time-invariant state fixed effect; τ_t is the survey year fixed effect; γ_c is the birth cohort fixed effect; and ε_{istc} is the error.

To construct the lifetime wage measure, we averaged the effective minimum wage in a child's state of residence for all ages from 0 until the survey year. We computed one version with nominal wages and one with real (or inflation-adjusted) wages. Given the absence of data on a household's movement in the NSCH and YRBSS, we assumed that a child remained in the same state since birth. In reality, 2–3% of households move between states in a typical year, per the American Community Survey, so our lifetime minimum wage variable was measured with some error. Even so, it allowed us to approximate the association between a child's cumulative exposure to minimum wage policies and their mental health later in childhood or adolescence.

As with the standard TWFE analyses, the lifetime minimum wage models allowed us to include all state-years of data, and their standard errors were clustered at the state level. Additionally, we applied the same Bonferroni corrections for multiple hypothesis testing.

Results. The lifetime minimum wage models provided mostly null, otherwise conflicting evidence for the association between long-term exposure to minimum wages and children's mental health (**eFigures 15–16**). Several estimates were significant at the 5% level, yet they pointed in different directions: Those in the NSCH were positively associated, yet those in the YRBSS,

negatively. Substantively similar outcomes also pointed in opposite directions, such as behavior problems and physical fights, or digestive issues and sadness (both symptoms of mood disorders). None remained statistically significant after Bonferroni corrections. Some CIs ruled out meaningfully large associations, although most were less precisely estimated.

Conclusions. Together, the lifetime minimum wage models did not provide consistent, compelling evidence that higher minimum wages sustained throughout a child's life were associated with better mental health later in childhood or adolescence. While some estimates provided suggestive evidence for specific outcomes, they pointed in conflicting directions.

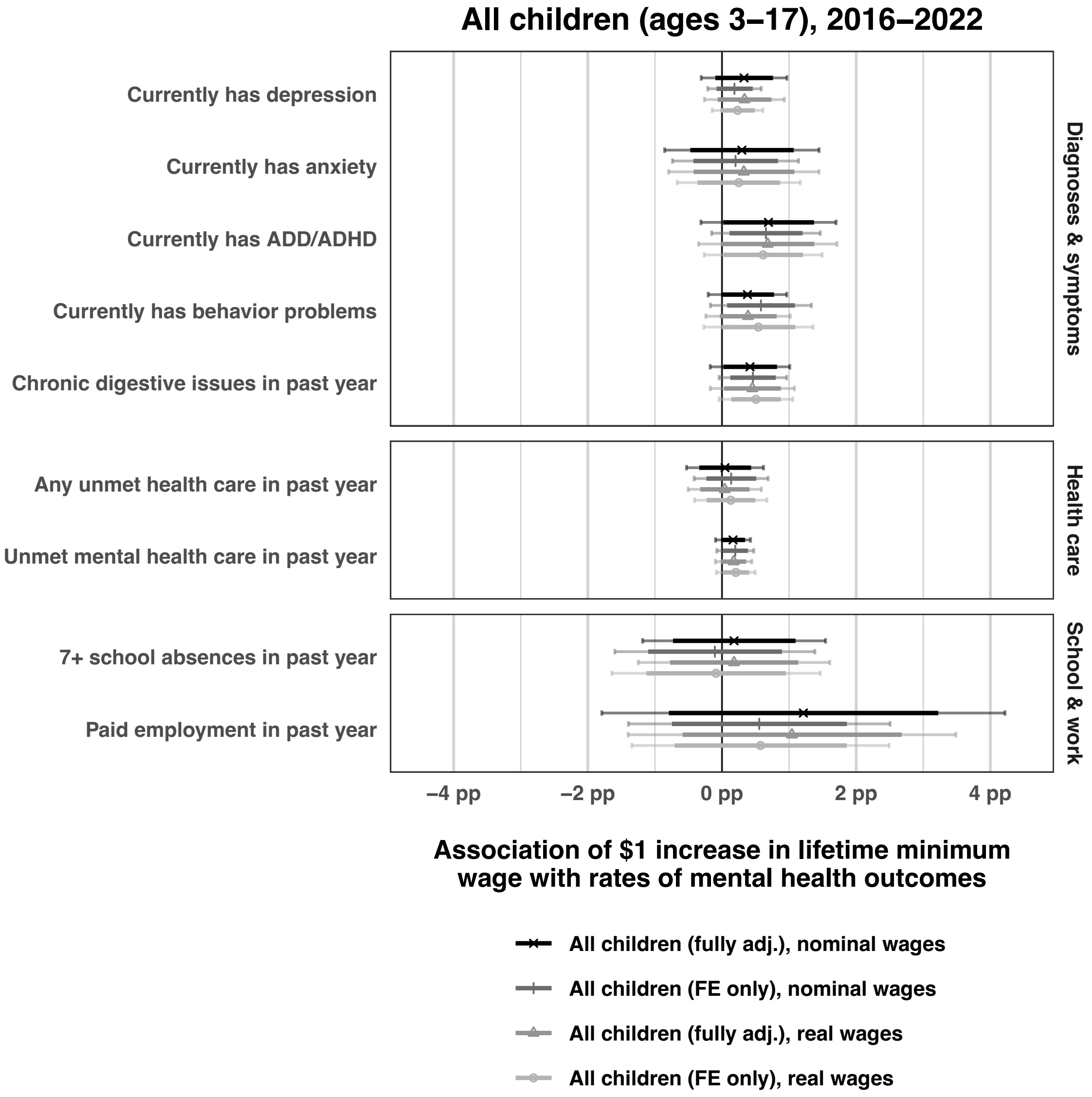

eFigure 15. Lifetime minimum wage models in the NSCH.

Notes: Based on OLS models using the average minimum wage to which a child was likely exposed throughout their life. Both nominal wages and real (inflation-adjusted) wages were tested. All models included state, year, and birth cohort fixed effects; fully adjusted models also included individual- and state-level controls per **eTable 3**. SEs were clustered at the state level. 95% CIs (thick) and 99.7% CIs for Bonferroni corrections (thin) are provided. N=185,433–238,826.

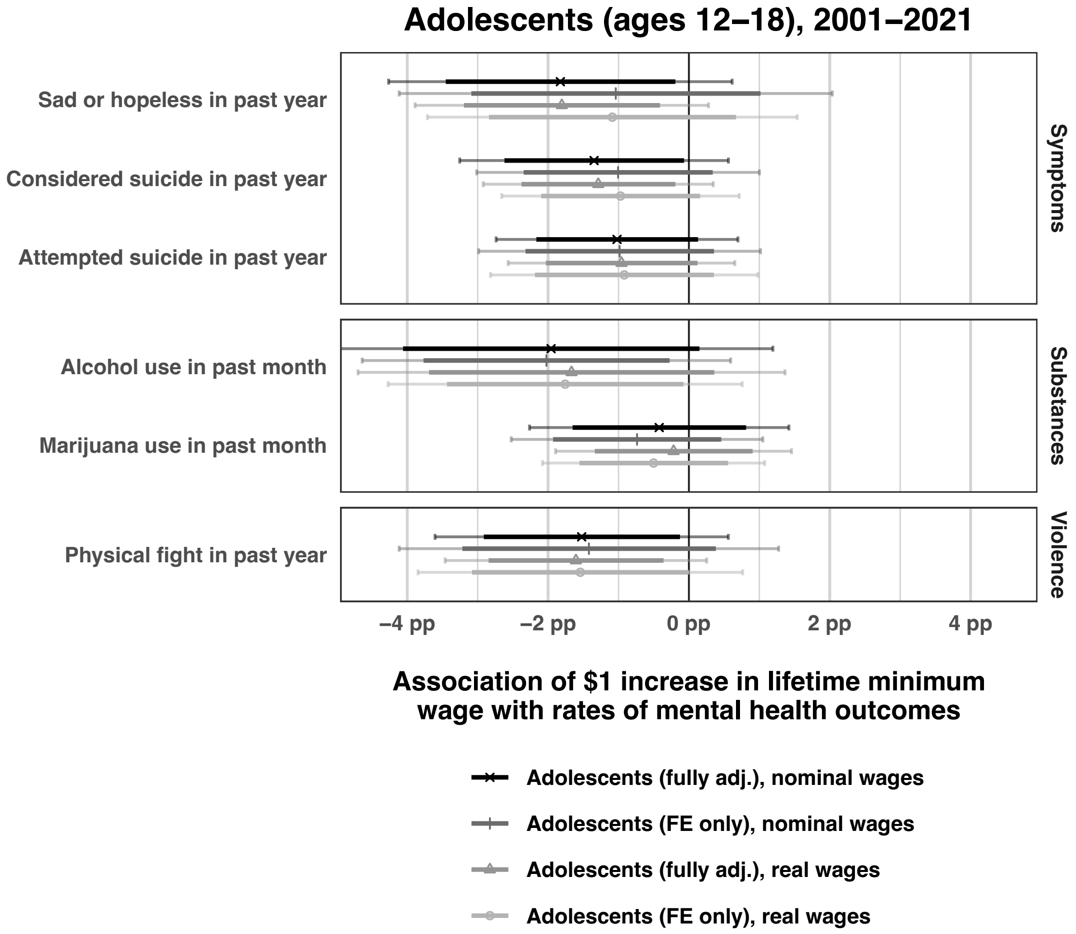

eFigure 16. Lifetime minimum wage models in the YRBSS.

Notes: Based on OLS models using the average minimum wage to which a child was likely exposed throughout their life. Both nominal wages and real (inflation-adjusted) wages were tested. All models included state, year, and birth cohort fixed effects; fully adjusted models also included individual- and state-level controls per **eTable 3**. SEs were clustered at the state level. 95% CIs (thick) and 99.7% CIs for Bonferroni corrections (thin) are provided. N=1,099,239–1,417,389.

eAppendix 10. Approach #3: Difference-in-Differences and Event Study Models

Motivation. Recent advancements in econometrics have highlighted the potential biases of TWFE models when units adopt policies at different times and experience dynamic treatment effects.[9–11] Consequently, our standard TWFE models may not have estimated unbiased causal effects. Given that unbiased estimates are valuable for policy makers and public health professionals, we implemented unbiased difference-in-differences with event studies as yet another approach for evaluating whether rising minimum waged affected children's mental health.

Methods. Difference-in-differences analyses require a group of treatment states with minimum wage variation over time and a group of control states without. The rise in the federal minimum wage from 2008–2010 complicated this search, so we limited our analyses to the years after. Then, we focused on the states that continued to use the federal minimum wage for several years, starting in 2010. This choice provided a cohort of states with identical minimum wage trajectories for many years and a washout period following the change in federal policy.

To define a set of treatment states, the challenge was balancing a suitably long pre-period for evaluating pre-trends and a suitably long post-period for evaluating long-term effects. To maximize the number of study years, we used the YRBSS. In choosing the YRBSS, however, we also had to balance concerns about YRBSS state-year missingness and statistical power.

We coded as treated the 10 states that raised their minimum wages above the federal minimum of $7.25 between the 2013 and 2015 waves of the YRBSS (**eFigure 17, panel A**). Meanwhile, the 20 states that remained at the federal minimum of $7.25 from 2010 to 2021 served as controls. All other states were excluded. This setup provided a 4-year pre-period (or 2 waves of the YRBSS) to evaluate for parallel pre-trends and a 7-year (or 4-wave) post-period to evaluate for long-run effects. By selecting states that raised their wages at the same time, we avoided the potential biases of staggered treatment timing in difference-in-differences models.[9–11]

During this period, treated states implemented a range of minimum wage increases, as shown in **eFigure 17, panel B**. Treated adolescents were exposed to a weighted mean wage of $10.20 in the post-period, or a $2.95 increase over baseline. Consequently, our difference-in-differences models estimated the average effect of a $2.95 increase in the minimum wage on the mental health of treated adolescents, rather than the $1 increase in the TWFE models.

The difference-in-differences models were specified as follows:

$$Y_{istc} = \beta_1(treated)_s + \beta_2(post2013)_t + \beta_3(treated \times post2013)_{st} +$$
$$\boldsymbol{\beta X_{istc}} + \boldsymbol{\beta Z_{st}} + \Delta_s + \tau_t + \gamma_c + \varepsilon_{istc}$$

where Y_{istc} is the mental health outcome for individual *i* in birth cohort *c* in state *s* in year *t*; $(treated)_s$ is an indicator for whether a state was in the treatment group (coded as 1) or control group (0) (shown in **eFigure 17, panel A**); $(post2013)_t$ is an indicator for whether an observation is in the pre-treatment period (2011 or 2013, coded as 0) or post-treatment period (2015 onward, 1); the coefficient on the interaction term, β_3, estimates the difference-in-differences, or the average treatment effect on treated adolescents; X_{istc} is a vector of individual-level controls (per **eTable 3**); Z_{st} is a vector of time-variant state-level policies (**eTable 3**); Δ_s is the time-invariant state fixed effect; τ_t is the year fixed effect; γ_c is the birth cohort fixed effect; and ε_{istc} is the error.

Additionally, we specified event study models, which were akin to the difference-in-differences models but allowed the treatment effect to vary by year. They let us visualize parallel pre-trends and evaluate how the effects changed over time. Treated adolescents were exposed to a mean minimum wage of $8.40 (or $1.15 over baseline) in 2015, $9.23 (or $1.98) in 2017, $10.89 (or $3.64) in 2019, and $11.56 ($4.31) in 2021 (**eFigure 18**). By 2021, treated states experienced a nearly 60% increase in mean minimum wages over control states. Because the treatment grew during this period, we should expect any effect on mental health to grow over time, too.

The event studies were estimated as follows:

$$Y_{istc} = \beta_1(treated)_s + \boldsymbol{\beta}(year)_t + \boldsymbol{\beta}(treated \times year)_{st} + \\ \boldsymbol{\beta X_{istc}} + \boldsymbol{\beta Z_{st}} + \Delta_s + \tau_t + \gamma_c + \varepsilon_{istc}$$

where we have a vector of coefficients of interest, one for the treatment effect in each year. The remaining terms mirror those for the difference-in-differences models (see above).

Together, the difference-in-differences and event study models allowed us to rigorously test the unbiased effect of raising the minimum wage on the mental health of adolescents. All models used the YRBSS weights and clustered SEs at the state level. We have provided 95% CIs and Bonferroni-corrected 99.2% CIs (since we only consider 6 outcomes, 0.05/6 = 0.0083, with critical values of 2.64). Models were estimated by OLS in the "lfe" package (v. 2.9–0) in R. The event study sensitivity analyses are described in **eAppendix 11** of **Supplement 1**.

<u>Results.</u> The main difference-in-differences models are presented in **eTable 7**, with the main event studies in **eFigure 19**. The outcomes included self-reported symptoms and coping mechanisms, which should be especially sensitive to changes in household financial stress.

However, these analyses provided largely null, otherwise conflicting evidence that rising minimum wages improved the mental health of adolescents. All adjusted difference-in-differences models were null, and many CIs ruled out meaningfully large improvements. Meanwhile, the event studies were inconsistent with the dynamics we would expect of a treatment that started in 2014–

2015 and grew in magnitude over time. Instead, the estimates moved in conflicting directions throughout the 7-year post-treatment period, with only occasional CIs suggesting meaningful, significant benefits in 2021. We find it implausible that treatment effects would suddenly manifest in 2021, or 6–7 years after the initial policy change, rather than gradually phase in throughout the post-period as minimum wages were gradually raised across treated states.

The event studies helped reduce concerns about non-parallel pre-trends, suggesting that the control states were an appropriate counterfactual for our treatment states. The one exception may have been physical fights, for which non-parallel deviations in the pre-trends suggest that our counterfactual may not have been fully appropriate for that outcome. (Even so, the models with a strictly balanced panel helped alleviate this concern as well; see **eAppendix 11**.)

Conclusions. Taken together, the difference-in-differences and event study analyses did not provide clear, compelling evidence that raising a state's minimum wage improved adolescents' mental health from 2011–2021. Even though occasional event study estimates were statistically significant after Bonferroni corrections, they were misaligned with policy changes and lacked the phased-in dynamics expected of steadily rising minimum wages in treated states.

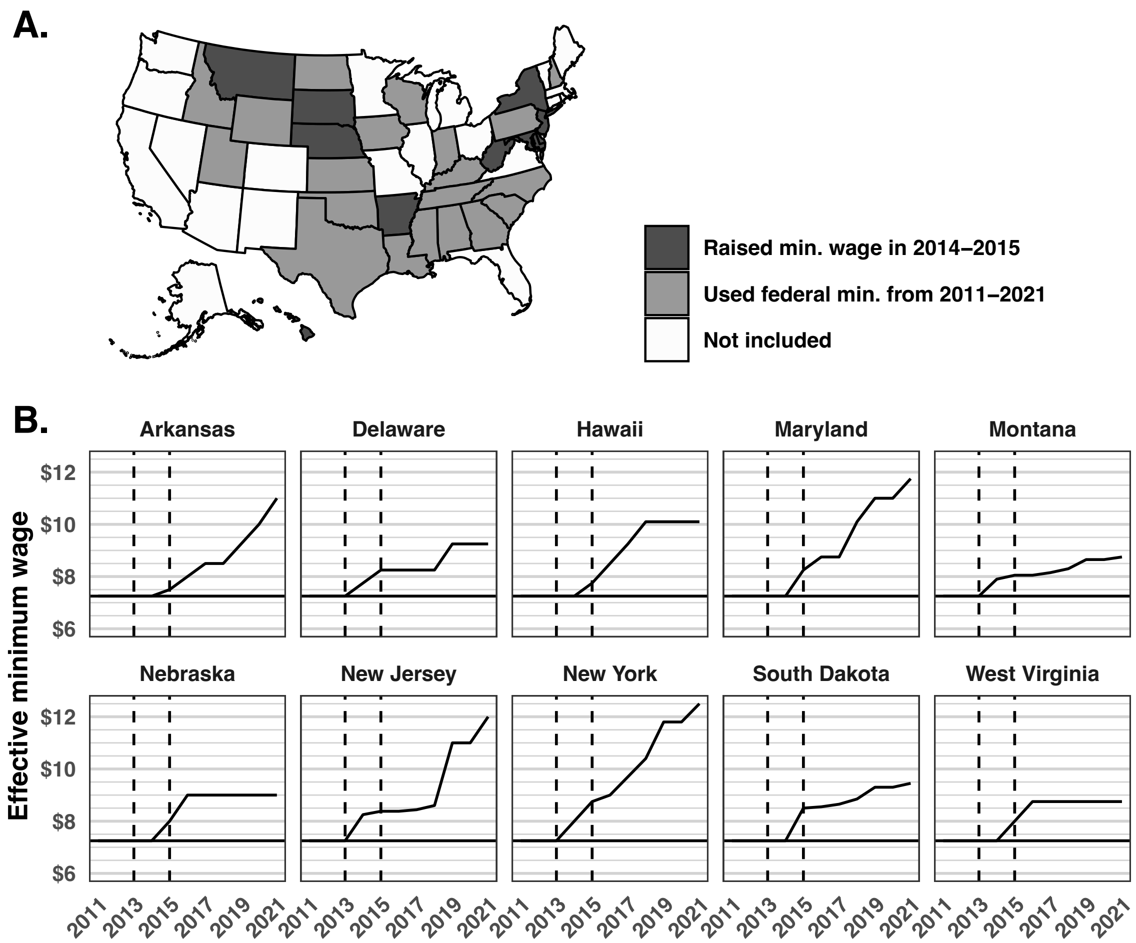

eFigure 17. Treatment and control states for the difference-in-differences models.

Notes: (A.) States that started raising their minimum wage above the federal minimum in 2014 or 2015 served as treatment states, while those that remained at the federal minimum served as controls. **(B.)** Effective minimum wages in treatment states, per U.S. Dept. of Labor data.

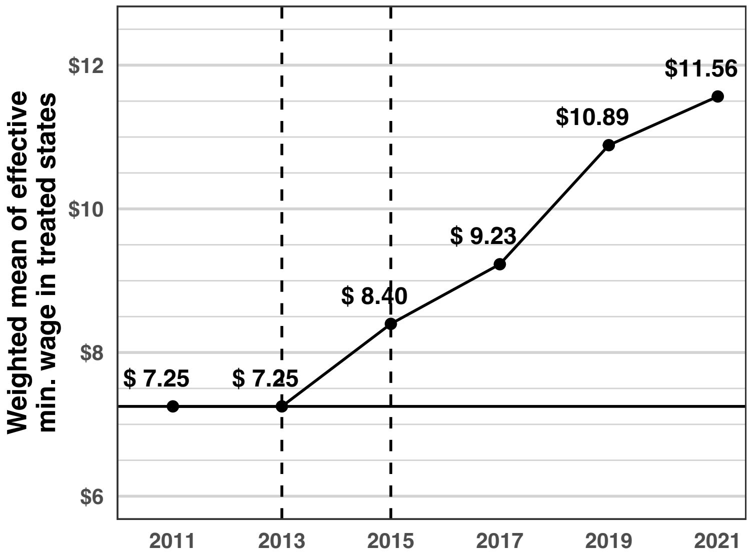

eFigure 18. Weighted mean minimum wage in treatment states by year.

Notes: These estimates provide the effective minimum wage to which the mean treated adolescent was exposed using the YRBSS weights. Based on U.S. Department of Labor data.

eTable 7. Difference-in-difference models in the YRBSS from 2011–2021.

	**Sad or hopeless in past year**		**Considered suicide in past year**	
	**FE only**	**Fully adj.**	**FE only**	**Fully adj.**
**Effect of raise in wage**	0.44 pp	0.25 pp	–0.24 pp	–0.19 pp
95% CIs	[–1.01, 1.88]	[–1.15, 1.66]	[–1.43, 0.96]	[–1.29, 0.90]
99.2% CIs	[–1.57, 2.44]	[–1.69, 2.20]	[–1.89, 1.42]	[–1.71, 1.33]
**Demographic controls**	No	Yes	No	Yes
**State policy controls**	No	Yes	No	Yes
**State, age, & birth cohort FEs**	Yes	Yes	Yes	Yes
**Cluster-robust SEs**	State	State	State	State
**Number of adolescents**	644,739	644,739	644,517	644,517
**Adjusted R2**	0.016	0.059	0.006	0.028

	**Attempted suicide in past year**		**Alcohol use in past month**	
	**FE only**	**Fully adj.**	**FE only**	**Fully adj.**
**Effect of raise in wage**	0.03 pp	0.22 pp	–0.98 pp	–0.85 pp
95% CIs	[–1.52, 1.58]	[–0.84, 1.29]	[–2.85, 0.88]	[–2.46, 0.76]
99.2% CIs	[–2.12, 2.18]	[–1.25, 1.69]	[–3.56, 1.59]	[–3.08, 1.38]
**Demographic controls**	No	Yes	No	Yes
**State policy controls**	No	Yes	No	Yes
**State, age, & birth cohort FEs**	Yes	Yes	Yes	Yes
**Cluster-robust SEs**	State	State	State	State
**Number of adolescents**	392,990	392,990	598,704	598,704
**Adjusted R2**	0.006	0.024	0.042	0.059

	**Marijuana use in past month**		**Physical fight in past year**	
	**FE only**	**Fully adj.**	**FE only**	**Fully adj.**
**Effect of raise in wage**	0.07 pp	–0.03 pp	0.51 pp	–0.32 pp
95% CIs	[–0.93, 1.06]	[–1.03, 0.96]	[–1.03, 2.06]	[–1.00, 1.64]
99.2% CIs	[–1.31, 1.44]	[–1.41, 1.35]	[–1.63, 2.65]	[–1.50, 2.15]
**Demographic controls**	No	Yes	No	Yes
**State policy controls**	No	Yes	No	Yes
**State, age, & birth cohort FEs**	Yes	Yes	Yes	Yes
**Cluster-robust SEs**	State	State	State	State
**Number of adolescents**	629,888	629,888	352,794	352,794
**Adjusted R2**	0.017	0.026	0.012	0.048

Notes: The coefficients provide the effect in percentage points (pp) of raising the state minimum wage above the federal minimum on adolescents' mental health from 2011–2021. The average raise was $2.95 over control states in the post-period. Based on OLS difference-in-differences models using the states in **eFigure 17**. All models included the indicated adjustments and fixed effects (see **eTable 3**). 95% CIs and 99.2% CIs for Bonferroni corrections are provided.

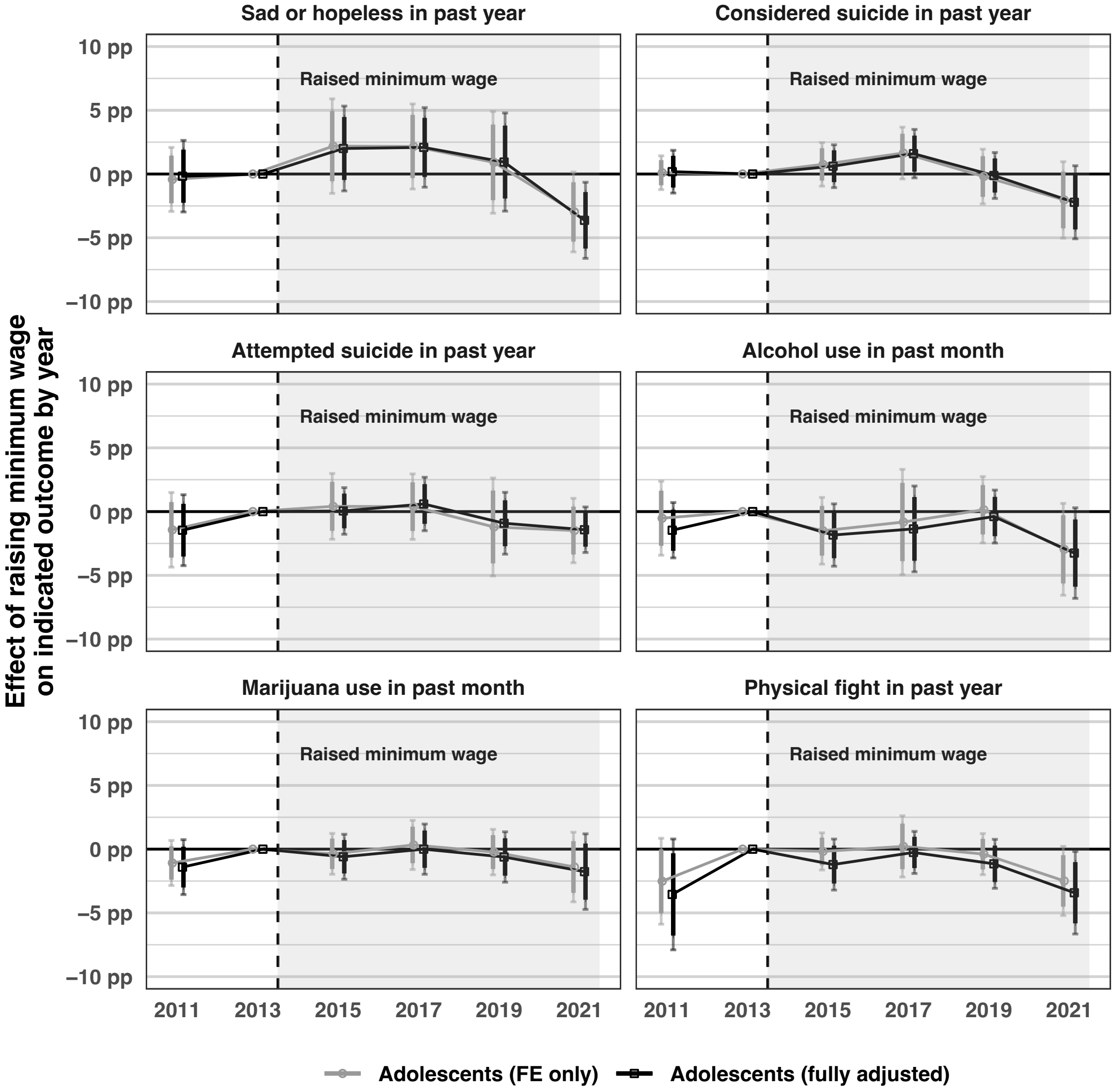

eFigure 19. Main event studies in the YRBSS from 2011–2021.

Notes: Each coefficient provides the effect of raising the minimum wage above the federal minimum wage in the indicated year. Average treatment wages by year are provided in **eFigure 18**. Based on OLS event study models using states that raised their minimum wages above the federal minimum in 2014 or 2015, compared to states that used the federal minimum for the entire analysis period. All models included state, age, and birth cohort FEs; fully adjusted models added individual- and state-level covariates per **eTable 3**. SEs are clustered at the state level. 95% CIs (thick) and 99.2% CIs for Bonferroni corrections (thin) are provided. N=352,794–644,739.

© 2024 Kavanagh NM et al. *JAMA Network Open.*

eAppendix 11. Approach #3: Event Study Sensitivity Analyses

We subjected the event studies to two sensitivity analyses:

Given the discussion of SEs in **eAppendix 8**, we reproduced the event study models using the YRBSS's nested clusters (**eFigure 20**). They were substantively similar to those with state-clustered SEs, suggesting that the inferences were not overly or underly conservative.

Next, since not all states fielded the YRBSS in all years, another concern might be that the main event studies were biased by an imbalanced panel. That is, the coefficients relied on different combinations of states depending on the year, which might have biased our inferences. To reduce this concern, we reproduced the models on strictly balanced panels of states; these panels varied by outcome since not all states fielded all questions in all years (**eTable 8**).

Like the main event studies, the balanced panel models were inconsistent with widespread improvements in adolescents' mental health (**eFigure 21**). Many coefficients were less precisely estimated given the smaller sample sizes, although the dynamics were similar. Notably, the pre-trends for physical fights were less worrisome than in the main event studies, suggesting that the control group in the strictly balanced panel provided a more appropriate counterfactual.

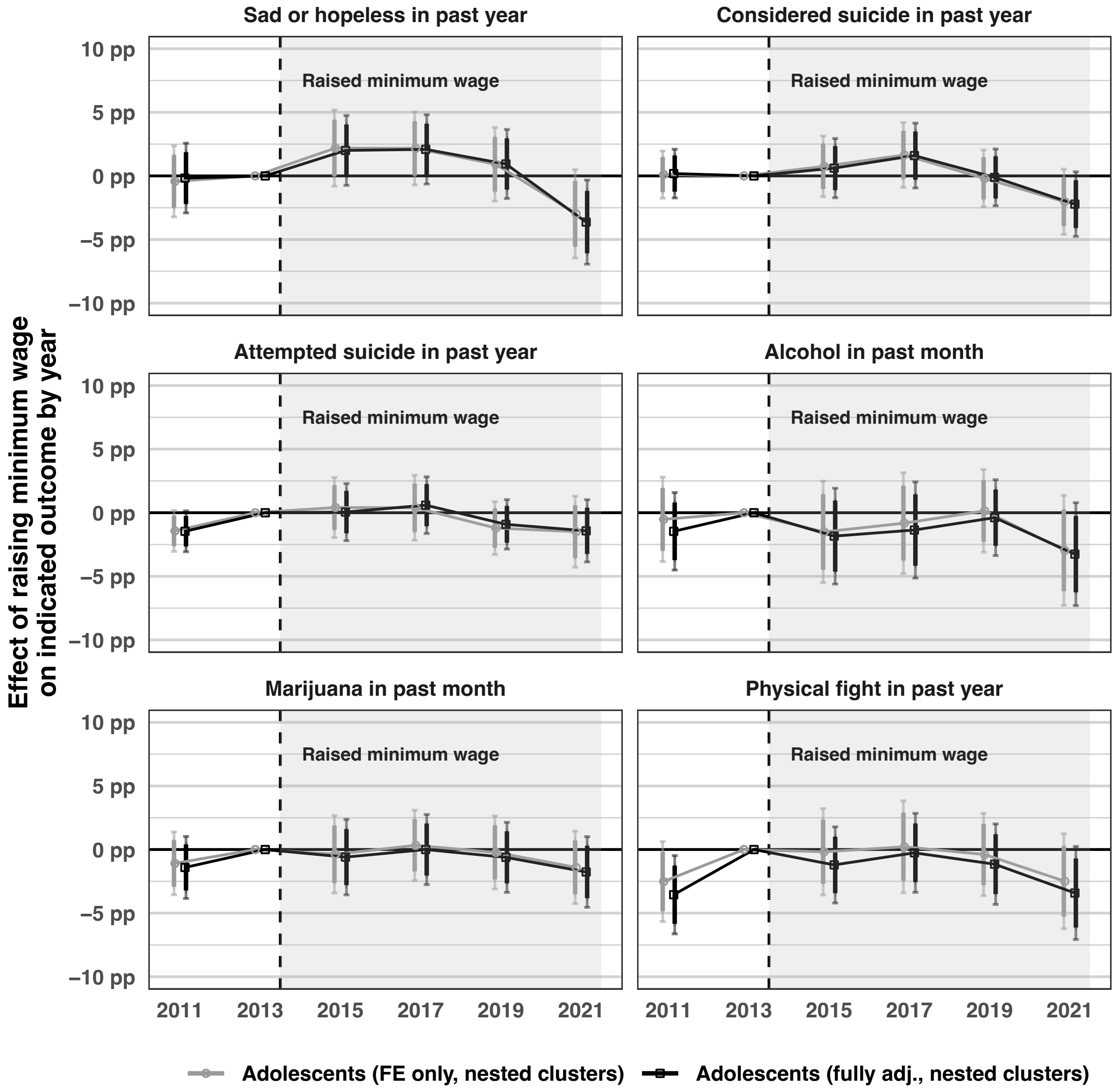

eFigure 20. Event studies using nested clusters in the YRBSS.

Notes: Re-estimation of the main event studies with SEs clustered per the YRBSS nested design. See **eFigure 19** for the state-clustered SEs. All models included state, year, and birth cohort FEs; fully adjusted models also included individual- and state-level covariates per **eTable 3**. 95% CIs (thick) and 99.2% CIs for Bonferroni corrections (thin) are provided. N=352,794–644,739.

eTable 8. States in balanced panels for event studies by outcome.

Outcome	Included states
**Sad or hopeless in past year**	**Treatment:** AR, HI, MD, MT, NE, NY, WV<br>**Control:** ID, KY, NC, ND, NH, OK, SC, TN
**Considered suicide in past year**	**Treatment:** AR, HI, MD, MT, NE, NY, WV<br>**Control:** ID, KY, NC, ND, NH, OK, TN
**Attempted suicide in past year**	**Treatment:** AR, HI, MT, NE, NY, WV<br>**Control:** ID, KY, ND, NH, OK, TN
**Alcohol use in past month**	**Treatment:** AR, HI, MD, MT, NE, NY, WV<br>**Control:** ID, KY, NC, ND, NH, OK, SC
**Marijuana use in past month**	**Treatment:** AR, HI, MD, MT, NE, NY, WV<br>**Control:** ID, KY, NC, ND, NH, OK, SC
**Physical fight in past year**	**Treatment:** AR, HI, MT, NE, NY, WV<br>**Control:** NC, SC

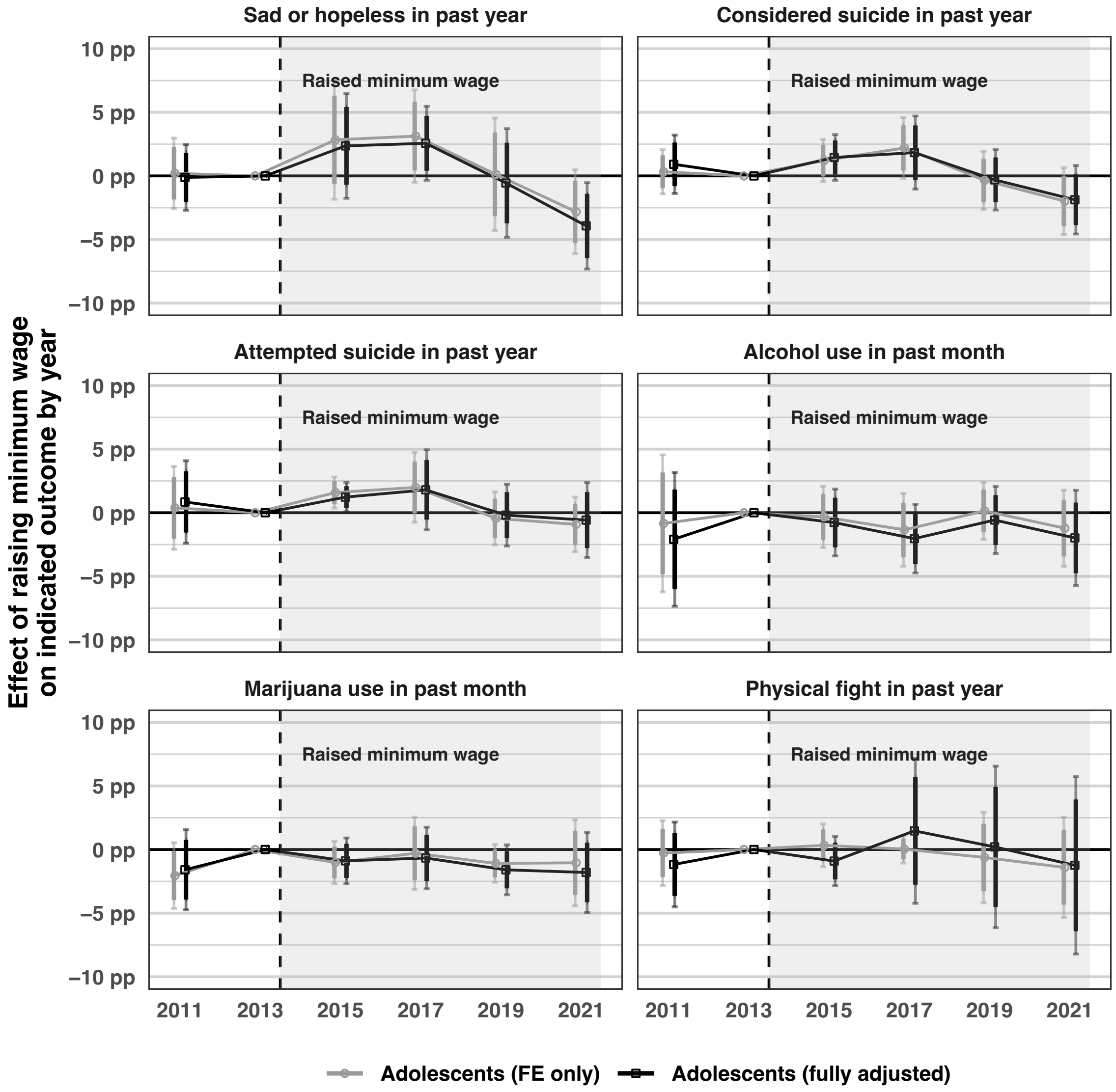

eFigure 21. Event studies using a strictly balanced panel in the YRBSS.

Notes: Re-estimation of the main event studies using strictly balanced panels of states (per **eTable 8**). All models included state, age, and birth cohort FEs; fully adjusted models also included individual- and state-level controls per **eTable 3**. SEs were clustered at the state level. 95% CIs (thick) and 99.2% CIs for Bonferroni corrections (thin) are provided. N=173,624–520,083.

eReferences

1. Averett SL, Smith JK, Wang Y. The effects of minimum wages on the health of working teenagers. *Applied Economics Letters*. 2017;24(16):1127-1130. doi:10.1080/13504851.2016.1259737

2. Averett SL, Smith JK, Wang Y. Minimum wages and the health of immigrants' children. *Applied Economics Letters*. 2021;28(11):894-901. doi:10.1080/13504851.2020.1784832

3. Wehby GL, Dave DM, Kaestner R. Effects of the Minimum Wage on Infant Health. *Journal of Policy Analysis and Management*. 2020;39(2):411-443. doi:10.1002/pam.22174

4. Wehby GL, Kaestner R, Lyu W, Dave DM. Effects of the Minimum Wage on Child Health. *American Journal of Health Economics*. 2022;8(3):412-448. doi:10.1086/719364

5. COUNCIL ON COMMUNITY PEDIATRICS, Gitterman BA, Flanagan PJ, et al. Poverty and Child Health in the United States. *Pediatrics*. 2016;137(4):e20160339. doi:10.1542/peds.2016-0339

6. Acharya A, Blackwell M, Sen M. Explaining Causal Findings Without Bias: Detecting and Assessing Direct Effects. *American Political Science Review*. 2016;110(3):512-529. doi:10.1017/S0003055416000216

7. Curran-Everett D. Multiple comparisons: philosophies and illustrations. *American Journal of Physiology-Regulatory, Integrative and Comparative Physiology*. 2000;279(1):R1-R8. doi:10.1152/ajpregu.2000.279.1.R1

8. Abadie A, Athey S, Imbens GW, Wooldridge JM. When Should You Adjust Standard Errors for Clustering? *The Quarterly Journal of Economics*. Published online October 6, 2022:qjac038. doi:10.1093/qje/qjac038

9. Goodman-Bacon A. Difference-in-differences with variation in treatment timing. *Journal of Econometrics*. 2021;225(2):254-277. doi:10.1016/j.jeconom.2021.03.014

10. Callaway B, Sant'Anna PHC. Difference-in-Differences with multiple time periods. *Journal of Econometrics*. 2021;225(2):200-230. doi:10.1016/j.jeconom.2020.12.001

11. Sun L, Abraham S. Estimating dynamic treatment effects in event studies with heterogeneous treatment effects. *Journal of Econometrics*. 2021;225(2):175-199. doi:10.1016/j.jeconom.2020.09.006
