# Supplementary material for: State Minimum Wage and Mental Health Among Children and Adolescents
Source: JAMA Netw Open. 2024 Oct 23;7(10):e2440810. doi: 10.1001/jamanetworkopen.2024.40810 (PMC11581516; doi:10.1001/jamanetworkopen.2024.40810)
Supplement: Supplement 2. — Data Sharing Statement [file jamanetwopen-e2440810-s002.pdf]

Data Sharing Statement

Kavanagh. State Minimum Wage and Mental Health Among Children and Adolescents. *JAMA Netw Open*. Published October 23, 2024. doi:10.1001/jamanetworkopen.2024.40810

Data
Data available: Yes
Data types: Deidentified participant data
How to access data: All de-identified data are publicly available at the Harvard Dataverse (https://doi.org/10.7910/DVN/HHZBSA).
When available: With publication

Supporting Documents
Document types: Statistical/analytic code
How to access documents: All replication code are publicly available at the Harvard Dataverse (https://doi.org/10.7910/DVN/HHZBSA).
When available: With publication

Additional Information
Who can access the data: To anyone.
Types of analyses: For any purpose.
Mechanisms of data availability: All replication materials are publicly available (https://doi.org/10.7910/DVN/HHZBSA).
